# *De novo* identification of mammalian ciliary motility proteins using cryo-EM

**DOI:** 10.1016/j.cell.2021.10.007

**Published:** 2021-10-28

**Authors:** Miao Gui, Hannah Farley, Priyanka Anujan, Jacob R. Anderson, Dale W. Maxwell, Jonathan B. Whitchurch, J. Josephine Botsch, Tao Qiu, Shimi Meleppattu, Sandeep K. Singh, Qi Zhang, James Thompson, Jane S. Lucas, Colin D. Bingle, Dominic P. Norris, Sudipto Roy, Alan Brown

**Affiliations:** 1 Department of Biological Chemistry and Molecular Pharmacology, Harvard Medical School, Boston, MA 02115, USA; 2 MRC Harwell Institute, Harwell Campus, Oxfordshire OX11 0RD, UK; 3 Institute of Molecular and Cell Biology, Proteos, 138673 Singapore, Singapore; 4 Department of Infection, Immunity & Cardiovascular Disease, The Medical School and The Florey Institute for Host Pathogen Interactions, University of Sheffield, Sheffield S10 2TN, UK; 5 School of Biological Sciences, University of Manchester, Manchester M13 9PT, UK; 6 Department of Genetics, Harvard Medical School, Boston, MA 02115, USA; 7 Biomedical Imaging Unit, Southampton General Hospital, Southampton, UK; 8 Primary Ciliary Dyskinesia Centre, NIHR Biomedical Research Centre, University Hospital Southampton NHS Foundation Trust, Southampton, UK; 9 University of Southampton Faculty of Medicine, School of Clinical and Experimental Medicine, Southampton, UK; 10 Department of Biological Sciences, National University of Singapore, 117543 Singapore, Singapore; 11 Department of Pediatrics, Yong Loo Ling School of Medicine, National University of Singapore, 1E Kent Ridge Road, 119288 Singapore, Singapore; 12 Present address: Medical School, Oxford University, Oxford OX3 9DU, UK; 13 Present address: Institute of Reproductive and Developmental Biology, Hammersmith Hospital, Imperial College, London, UK; 14 Present address: Department of Molecular Machines and Signaling, Max Planck Institute of Biochemistry, 82152 Martinsried, Germany; 15 These authors contributed equally; 16 Lead contact

## Abstract

Dynein-decorated doublet microtubules (DMTs) are critical components of the oscillatory molecular machine of cilia, the axoneme, and have luminal surfaces patterned periodically by microtubule inner proteins (MIPs). Here we present an atomic model of the 48-nm repeat of a mammalian DMT, derived from a cryoelectron microscopy (cryo-EM) map of the complex isolated from bovine respiratory cilia. The structure uncovers principles of doublet microtubule organization and features specific to vertebrate cilia, including previously unknown MIPs, a luminal bundle of tektin filaments, and a pentameric dynein-docking complex. We identify a mechanism for bridging 48- to 24-nm periodicity across the microtubule wall and show that loss of the proteins involved causes defective ciliary motility and laterality abnormalities in zebrafish and mice. Our structure identifies candidate genes for diagnosis of ciliopathies and provides a framework to understand their functions in driving ciliary motility.

## In brief

Characterizing the structural organization of mammalian ciliary microtubules provides a context for understanding cilium dynamics and the potential effect of mutations associated with human ciliopathies.

## Graphical Abstract

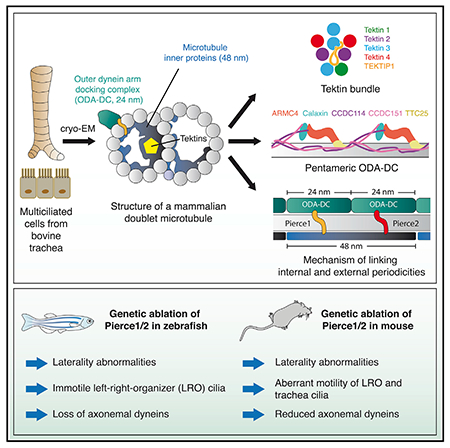

## INTRODUCTION

Motile cilia are eukaryotic organelles responsible for cellular locomotion and movement of extracellular fluids. For example, during vertebrate embryogenesis, motile cilia are responsible for the directional flow of extraembryonic fluids within the left-right organizer (LRO) that establishes left-right asymmetry of visceral organs like the heart (Nonaka et al., 1998). In adult vertebrates, motile cilia power the movement of spermatozoa and the flow of mucus in the respiratory system (reviewed in Zhou and Roy, 2015). Consistent with these varied functions, impairment of ciliary motility can cause laterality abnormalities, including congenital heart defects, infertility, and chronic respiratory disease, that often collectively manifest in the ciliopathy primary ciliary dyskinesia (PCD) (Legendre et al., 2021).

The beat of motile cilia is generated by the axoneme, a circular arrangement of dynein-decorated doublet microtubules (DMTs). The axonemes of most motile cilia have a “9+2” architecture, with nine DMTs surrounding a pair of singlet microtubules. A cryoelectron microscopy (cryo-EM) study of DMTs from the biflagellate alga *Chlamydomonas reinhardtii* revealed that their luminal surfaces are patterned by a 48-nm repeating network of at least 33 different microtubule inner proteins (MIPs) (Ma et al., 2019). MIPs are a universal feature of DMTs (Imhof et al., 2019; Nicastro et al., 2011; 2006), but electron cryotomography (cryo-ET) has shown that the MIPs in mammalian cilia (Greenan et al., 2020) differ visually from those in *Chlamydomonas* flagella (Ma et al., 2019).

Here we set out to answer three questions relating to mammalian ciliary architecture and its aberrancy in ciliopathies. First, which MIPs are present in mammalian DMTs, and do MIPs conserved across eukaryotic lineages reveal principles of axonemal organization? Second, how do the force-generating dynein motors of the axoneme, known as the outer dynein arms (ODAs), attach to DMTs with 24-nm periodicity? Recent cryo-EM structures have shown that algae and protozoan ciliates utilize a trimeric docking complex (the ODA-DC) (Kubo et al., 2021; Walton et al., 2021), but the DC3 subunit of this complex is not found in mammals (Casey et al., 2003). Third, what unites the MIP architecture and the exterior ODAs, and is this interconnectivity important for ciliary motility and function? In zebrafish and mice, genetic ablation of the suspected MIPs CFAP53 (Ide et al., 2020; Narasimhan et al., 2015; Noël et al., 2016) and MNS1 (Zhou et al., 2012) causes loss of ODAs from axonemes and disrupts ciliary motility. Despite evidence showing that MIPs are important for the physiologic positioning of ODAs, the molecular mechanism by which this is achieved is unknown.

To address these questions, we used cryo-EM to build an atomic model of DMTs isolated from bovine respiratory cilia, a paradigm for all motile cilia with 9+2 axonemes. The structure identifies MIPs specific to vertebrate axonemes, a pentameric ODA-DC, and a mechanism that links the internal and external periodicities that, when disrupted genetically, causes impaired ciliary motility in the LRO and alteration in the chirality of visceral organs. Our work reveals organizational principles of ciliary axonemes and provides a reference to improve the diagnosis and molecular understanding of ciliopathies. The ability to implicate new proteins in ciliary biology and, potentially, human disease, further establishes cryo-EM as an important tool for reverse genetics in vertebrate biology.

## RESULTS

### Structure determination

**Figure 1.**
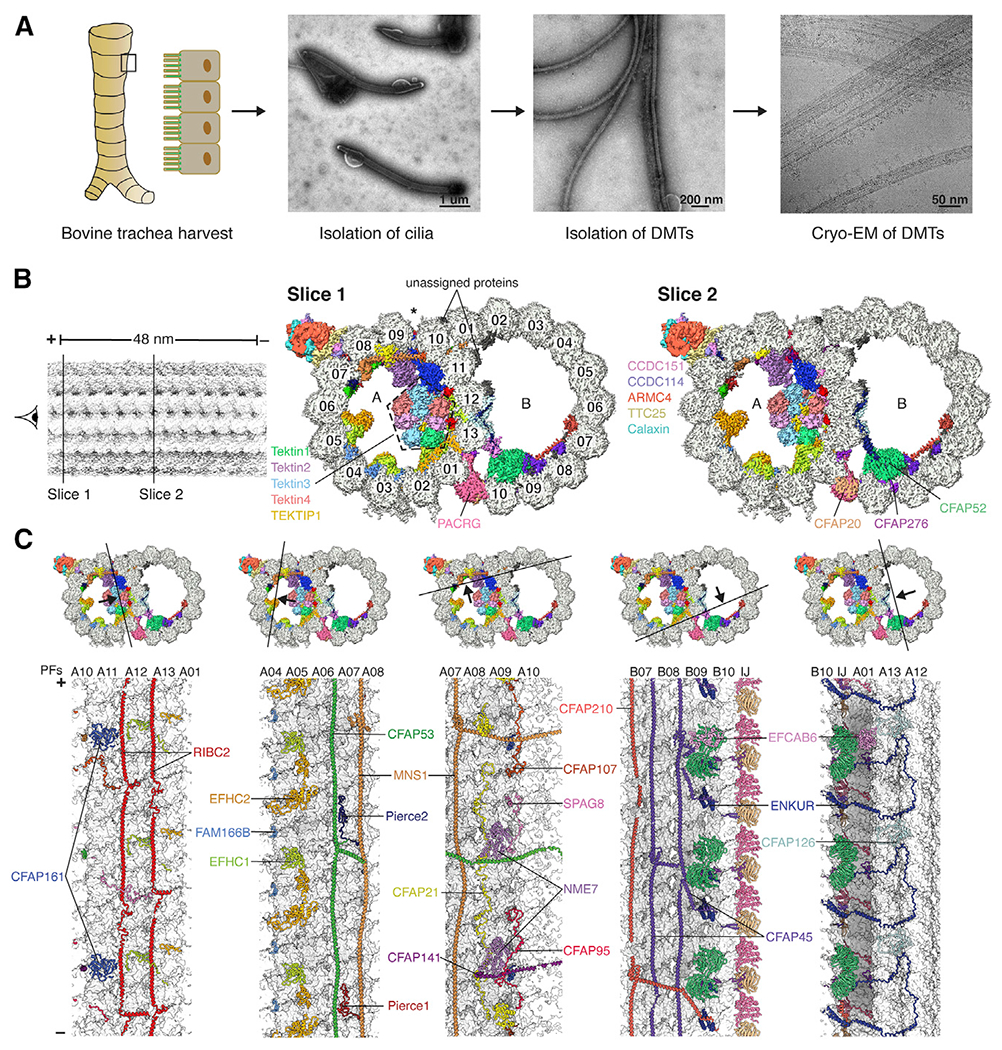
The 48-nm repeat structure of bovine doublet microtubules (DMTs) (A) Isolation of bovine DMTs for cryo-EM analysis. (B) Two slices through the DMT map, showing density for the MIPs and ODA-DC. Protofilaments are numbered, and the seam of the A tubule is marked with an asterisk. MIP labeling continues in (C). (C) The cross sections (top) show the DMT map colored by subunit, and the longitudinal sections (bottom) show the models of the MIPs, with tubulin in surface representation. Tektins are omitted for clarity in the longitudinal sections. PF, protofilament; IJ, inner junction. In (B) and (C), the minus (−) and plus (+) ends of the DMT are indicated. See also Figures S1 and S2, Tables S1 and S2, and Video S1.

To obtain mammalian DMTs for structural characterization, we mechanically dislodged motile cilia from bovine tracheae, detergent-solubilized their ciliary membranes, and dissociated their axonemes into DMTs (Figure 1A) prior to analysis by mass spectrometry (Table S1) and cryo-EM (Figure 1; Figure S1). Five cryo-EM datasets (Table S2) were processed to determine the structure of the 48-nm repeat of the bovine DMT (Figure S1; Methods S1) to a nominal resolution of 3.4 Å (Figure S1B) and a local resolution range of 3.2–4.0 Å (Figure S1D). A map of the 96-nm external repeat confirmed the overall periodicity of the MIP structure as 48 nm (Figure S1E), and comparison with the subtomogram average of bovine DMT (Greenan et al., 2020) showed that all prominent MIPs were retained (Figure S1F).

### Universal and mammal-specific features of DMTs

**Figure 2.**
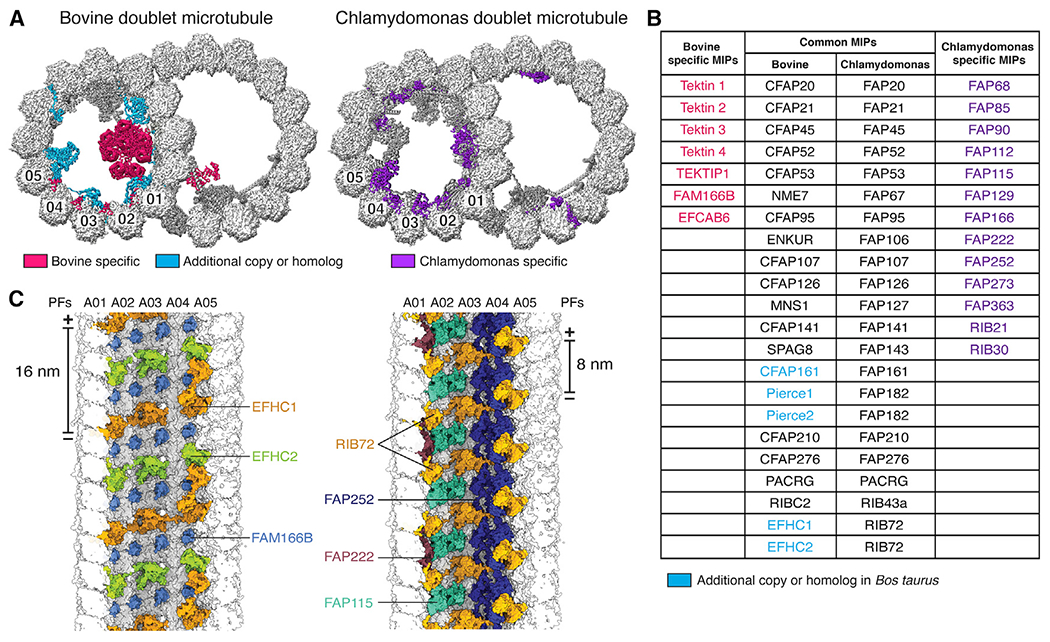
Microtubule inner proteins (MIPs) of mammalian DMTs (A) Cross-sections of bovine (left) and *Chlamydomonas* (right) DMTs, with MIPs colored by conservation. MIPs present in both organisms are colored dark gray. (B) Table of MIPs identified in bovine and *Chlamydomonas* DMTs. Protein names are colored according to their classification in (A). The names of shared MIPs are given for bovine and *C. reinhardtii*. (C) View from the A-tubule lumen showing bovine (left) and *Chlamydomonas* (right) MIPs bound to PFs A01–A05. *Chlamydomonas* RIB72 repeats every 8 nm, whereas its two bovine paralogs, EFHC1 and EFHC2, repeat every 16 nm. See also Video S1.

The well-resolved maps enabled building of an atomic model (Figures 1B and 1C; Table S2; Video S1). Of the identified 29 MIPs (Figure 2B; Methods S1), 22 have orthologs in the atomic model of the *Chlamydomonas* DMT (Ma et al., 2019; Figure 2). The seven additional MIPs include four tektins (described below), C19orf71, EFCAB6, and FAM166B (Figures 2A and 2B). In addition to these distinct MIPs, the mammalian DMT contains paralogs of single MIPs found in *Chlamydomonas*–two paralogs each for FAP182 (Pierce1 and Pierce2) and RIB72 (EFHC1 and EFHC2)–and an additional copy of CFAP161. The majority of the 13 MIPs present in *Chlamydomonas* but not bovine DMTs (listed in Figure 2B) fall into two major classes: those that overlap with the binding site for tektin in the bovine structure (FAP166, FAP22, FAP273, FAP363, RIB21, and RIB30) and those that have putative calcium-binding domains (FAP85, FAP115, FAP252, and RIB30). The additional calcium-binding proteins in *Chlamydomonas* may indicate additional mechanisms of calcium regulation specific to the motility of algal flagella.

Comparison of MIPs present in algal (Ma et al., 2019), ciliate (Khalifa et al., 2020), and mammalian DMTs revealed three organization principles. First, MIP architectures have 48-nm periodicity, but individual MIPs can have 8-, 16-, and 48-nm periodicities, and their distribution within DMTs can vary (Figure S2A). Bovine DMTs have a much larger region of 16-nm periodicity than in *Chlamydomonas* because of the presence of tektins and additional paralogs of RIB72 in the A tubule. Second, the MIP architecture is in register with the external 24- and 96-nm repeats despite no MIPs having these periodicities. Below we identify a mechanism that links the 24- and 48-nm periodicities, but mechanisms that link the 48- and 96-nm periodicities remain unclear. Third, the MIP organization at the inner and outer junctions is conserved across algae, ciliates, and mammals (Figures S2B–S2D), suggesting that proteins at these locations mediate the assembly and stability of the DMT architecture. The only major addition at the inner junction in mammals is EFCAB6, which has multiple EF-hand motifs and may therefore perform a role similar to that of the alga-specific calcium-binding proteins.

### Hyperstable tektin filaments occupy the A-tubule lumen

**Figure 3.**
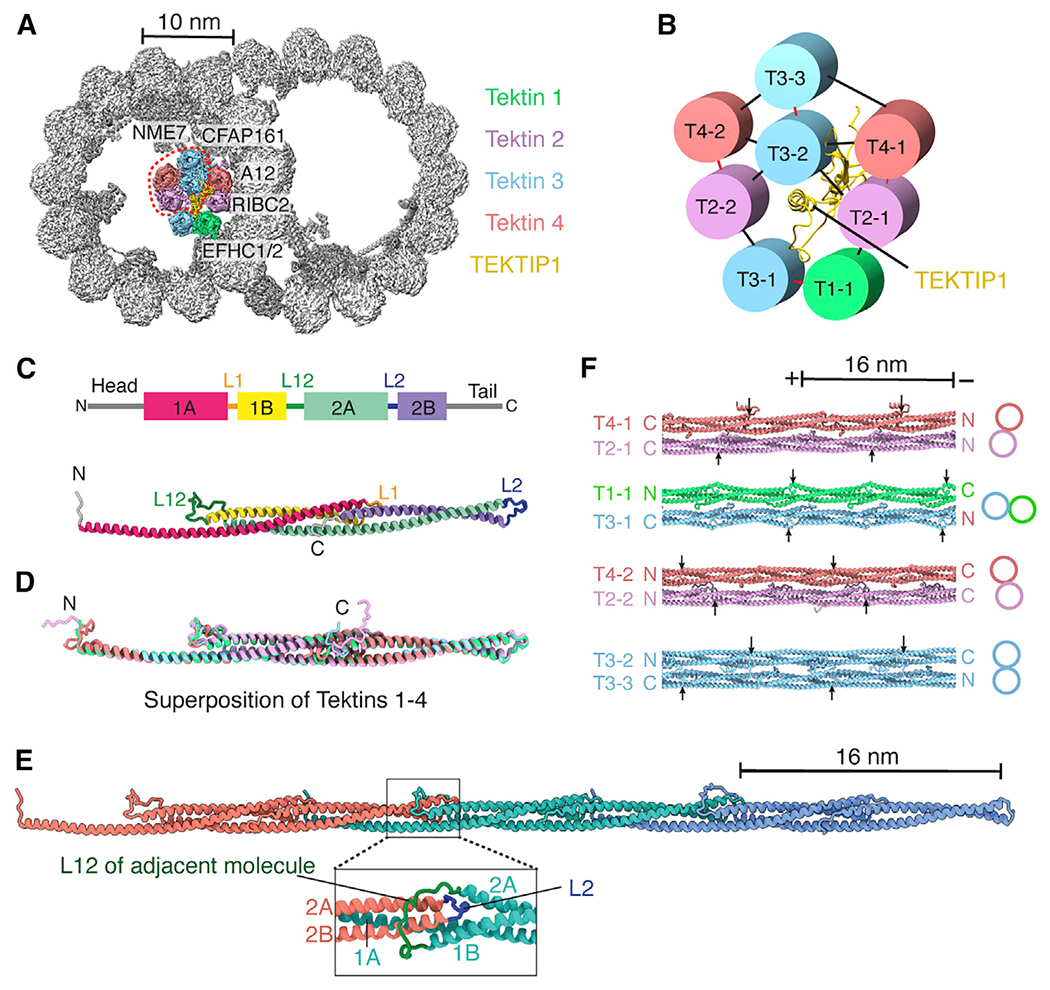
Tracheal DMTs contain a luminal bundle of tektin filaments (A) Cross-section of the bovine DMT, showing a luminal bundle of tektin filaments. The luminal-most tektin filaments (circled) are absent from some particles. The MIPs and PF A12 that interact with tektins are labeled. (B) Interaction network showing the pentagonal organization of tektin paralogs and TEKTIP1. Intradimer contacts are shown in red, and interdimer contacts are shown in black. (C) Secondary structure profile (top) and tertiary structure (bottom) of a tektin 1 monomer. (D) Superposition of tektin paralogs. The conserved rod domains superpose with a root-mean-square deviation (RMSD) of 1.0–1.7 Å. (E) Quaternary structure of a tektin filament. The expanded view shows the L12 loop clamping around the L2 loop of an adjacent molecule. (F) Interactions between tektin dimers as they occur in a section of the 8-tektin bundle. Filament orientation is indicated by labeling their N and C termini. Arrows mark the start of helix 1A in each tektin molecule to show the offsets in their registry. The two T2:T4 heterodimers are identical except that their N and C termini adopt different conformations because of the interactions they make within the bundle. See also Figures S3 and S4, Table S1, and Video S1.

A major difference between mammalian and algal DMTs is the presence of a bundle of helical tektin filaments within the A-tubule lumen (Figure 3A; Video S1). Tektins are among the most abundant and conserved ciliary proteins. They form hyperstable polymers (Linck et al., 1982) that are proposed to resemble intermediate filaments (Amos, 2008). Despite their importance for sperm motility (Roy et al., 2007; 2009; Tanaka et al., 2004), the structure, position, higher-order assembly, and function of tektins is controversial, in part because the number of tektin paralogs varies greatly between species (Bastin and Schneider, 2019), and tektins have been localized to both tubules (Linck, 1976; Linck and Langevin, 1982; Yanagisawa and Kamiya, 2004) and even outside of the axoneme (Iida et al., 2006; Murayama et al., 2008; Takiguchi et al., 2011).

Our structure conclusively places bovine tektins at the ribbon (protofilaments A10-A01) of the A tubule, which is consistent with their expected location in sea urchin sperm (Linck, 1976; Linck and Langevin, 1982) but inconsistent with their localization to the *Chlamydomonas* B tubule (Yanagisawa and Kamiya, 2004). Three-dimensional classification reveals that the bovine tektin bundle contains four or eight tektin filaments (Figure 3A; Methods S1). The four-tektin bundle may result from loss of the innermost tektins during purification because electron micrographs of human sperm flagella show a pentagonal structure resembling the full bundle on each of the nine DMTs (Afzelius et al., 1995). The eight-tektin bundle contains four of the five mammalian tektin paralogs (tektin 1–4); tektin 5 expression appears to be restricted to sperm cells (Murayama et al., 2008; Uhlén et al., 2015). Because chemical crosslinking has shown that sea urchin tektins form homo- and heterodimers (Pirner and Linck, 1994), we interpret the pentagonal architecture of the eight-tektin bundle as being formed by four pairs of dimers (Figure 3B), with each dimer maintained predominantly by polar interactions. Only tektin T4-1 interacts directly with the microtubule surface, indicating that tektin positioning may be dependent on prior association of other MIPs with the DMT (Figure 3A; Video S1). Additional ribbon-bound MIPs would sterically hinder tektin binding in the same position in *Chlamydomonas* DMTs (Ma et al., 2019).

At the center of the tektin bundle is C19orf71 (Figure 3B), a relatively unstructured protein we identified by denaturing the microtubule and performing mass spectrometry on the tektin-enriched sample (Table S1). Given the central position of C19orf71, we renamed it tektin bundle interacting protein 1 (TE-KIP1) and hypothesize that it may function to recruit tektins or stabilize the bundle.

Each individual tektin filament is formed by monomers stacked head to tail every 16 nm, consistent with calculations from sea urchin sperm (Pirner and Linck, 1994). Each monomer has a rod domain of four helices (1A, 1B, 2A, and 2B) separated by three linkers (L1, L12, and L2) (Figure 3C). Each helix folds back on the preceding helix in a concertina-like fashion (Figures 3C and 3D). Because helices 1A and 2A are twice the length of helices 1B and 2B, this generates a central three-helix bundle with helical overhangs that pair with the overhangs of adjacent monomers to create a continuous filament (Figure 3E). These patterns of alternating helix length and concertina folding are conserved in different tektin paralogs and throughout evolution (Figure 3D; Figure S3). Helices 1A and 2A are always approximately 108 residues (Figure S3B), consistent with all tektins having 16-nm repeat lengths. The three linkers within the rod domain are also highly conserved (Figure S3A; Amos, 2008; Bastin and Schneider, 2019) and play an important role in filament formation by stabilizing head-to-tail interactions (Figure 3E). Despite the conservation of sequence and helix length, there is remarkable diversity in how tektin filaments assemble. The filaments in a dimer and the dimers within a bundle can be oriented in parallel or antiparallel configurations, and the registers of their 16-nm repeats are offset (Figure 3F). The diversity in tektin composition, orientation, and register may contribute to the stability of the tektin bundle that allows them to resist chemical denaturation (Linck et al., 1982).

### Mammals have a pentameric ODA-DC

**Figure 4.**
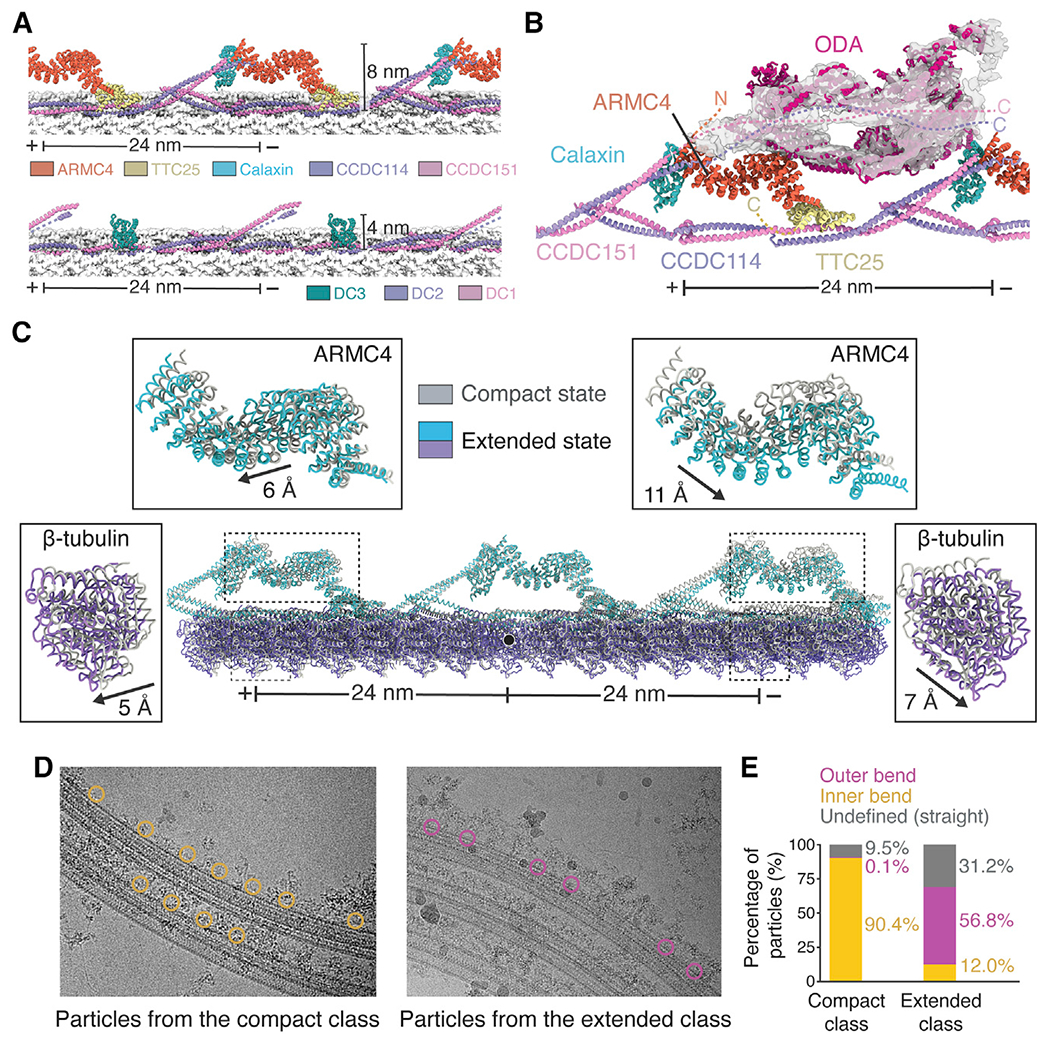
Structure and dynamics of the mammalian outer dynein arm (ODA) and ODA docking complex (ODA-DC) (A) Models of bovine (top) and *Chlamydomonas* (bottom) ODA-DCs. Tubulin is shown in surface representation. (B) Model of the *Chlamydomonas* ODA (PDB: 7KZM) docked into the cryo-EM map of the bovine ODA. Dashed lines represent the predicted locations of the termini of ODA-DC subunits when ODA is bound. (C) Analysis of tubulin lattice spacing reveals an extended and compact conformation with different ODA-DC conformations. The two models are superposed on the central tubulin (marked with a black circle). The displacement of ARMC4 and β-tubulin between classes was calculated using the mass center of the molecules. (D) Particles from the compact and extended classes mapped back onto the micrographs. (E) Quantification of the particle locations observed in (D). Only micrographs with 8 or more particles were analyzed. Compact class, n = 1,017; extended class, n = 1,201. See also Figure S4 and Video S2.

Our cryo-EM maps revealed the mammalian ODA-DC as a wave-like structure with 24-nm periodicity on the outside of protofilaments A07 and A08 (Figure 4A). Compared with the trimeric ODA-DC found in algae (Ma et al., 2019; Walton et al., 2021; Figure 4A; Figure S4A; Video S2A), the larger mammalian ODA-DC extends twice as far from the DMT surface. We exploited the 24-nm periodicity to determine a higher-resolution map of the ODA-DC than could be obtained from the 48-nm data (Figure S1; Methods S1). The improved map quality allowed identification of five subunits: CCDC114, CCDC151, ARMC4, TTC25, and Calaxin (renamed from EFCAB1) (Figure 4B). The first four of these were expected components of the ODA-DC because their absence causes ODA loss and PCD (Onoufriadis et al., 2013; Hjeij et al., 2013; 2014; Wallmeier et al., 2016). CCDC114 and CCDC151 form a coiled coil within the A07/A08 interprotofilament cleft, analogous to the DC1/2 coiled coil of the trimeric ODA-DC. The tetratricopeptide repeats of TTC25 and the armadillo repeats of ARMC4 form an α-solenoid bridge between neighboring copies of CCDC114/151. The arrangement of subunits within the ODA-DC explains periodicity measurements of TTC25 from immunoelectron microscopy studies (Ogawa and Inaba, 2006); co-immunoprecipitation of CCDC114 with TTC25 (Wallmeier et al., 2016); dependency of ARMC4 localization on CCDC114 (Hjeij et al., 2013); dependency of CCDC151, CCDC114, and ARMC4 on TTC25 (Wallmeier et al., 2016); and why CCDC114 localization is unaffected in ARMC4 mutants (Hjeij et al., 2013).

The fifth subunit of the ODA-DC, Calaxin, binds at the interface between ARMC4 and the distal CCDC114/151 (Figures 4A and 4B). Although not assigned previously as a component of the ODA-DC, *Ciona* Calaxin copurifies with ODAs (Hozumi et al., 2006) and localizes on the axoneme in the vicinity of the ODA by immunogold labeling (Mizuno et al., 2009). Unlike the other ODA-DC subunits that are essential for proper ODA placement, Calaxin may only have a regulatory role because sea urchin Calaxin morphants have impaired sperm locomotion despite apparently normal distributions of ODAs (Mizuno et al., 2017). Regulation of ciliary motility by Calaxin may be calcium dependent because bovine Calaxin has four EF-hand motifs (Shojima et al., 2018), and *Ciona* Calaxin suppresses ODA-driven microtubule sliding *in vitro* at high calcium concentrations (Mizuno et al., 2012).

### Basis for ODA attachment to DMTs

To determine how the ODA-DC contributes to the docking of ODAs to the DMT, we used three-dimensional classification to isolate a subset of dynein-bound particles (Figure S1; Methods S1). An 8-Å-resolution map reconstructed from these particles revealed that ARMC4 is the major dynein-binding subunit (Figure 4B), consistent with near-complete loss of ODAs in respiratory cilia from individuals with PCD with mutations in ARMC4 (Hjeij et al., 2013; Onoufriadis et al., 2014). We speculate that other ODA-DC subunits, especially CCDC114/151, also interact directly with the ODA through flexible regions not resolved in the ODA-free map (Figure 4B). This phenomenon of ordering upon binding also occurs in algae and ciliates, where the C termini of DC1 and DC2 only become structured in the presence of dynein (Kubo et al., 2021; Walton et al., 2021). There is also evidence from crosslinking that Calaxin can interact with the innermost dynein in a calcium-dependent manner (Mizuno et al., 2009). Higher-resolution structures of a microtubule-bound mammalian ODA will be required to determine the molecular details of these interactions.

### ODA-DC conformation changes with microtubule curvature

Using three-dimensional classification (Figure S1; Methods S1), we observed that the protofilaments that bind the ODA-DC (A07-A08) adopt compact and extended forms with distinct lattice dimensions (Figure 4C; Video S2B). We mapped the particles within these classes back onto the micrographs (Figure 4D) and found that particles with an extended lattice came predominantly from the outer bend, and particles with a compact lattice came mostly from the inner bend (Figure 4E). Thus, differences in tubulin longitudinal spacing are correlated with microtubule curvature. The ODA-DC adopts different conformations in these two states (Figure 4C; Video S2B), providing a putative mechanism by which the DC could regulate ODA conformation in response to bending of the axoneme during ciliary motility (Discussion). Although our low particle number (n = 8,755) prevented a similar analysis with ODA-bound DMTs, we did observe large-scale conformational changes in ODAs by multi-body analysis that could correspond to the curvature induced changes predicted by this model (Video S2C; Methods S1).

### Paralogous wall-spanning proteins link the external ODA-DC to the internal MIP structure

**Figure 5.**
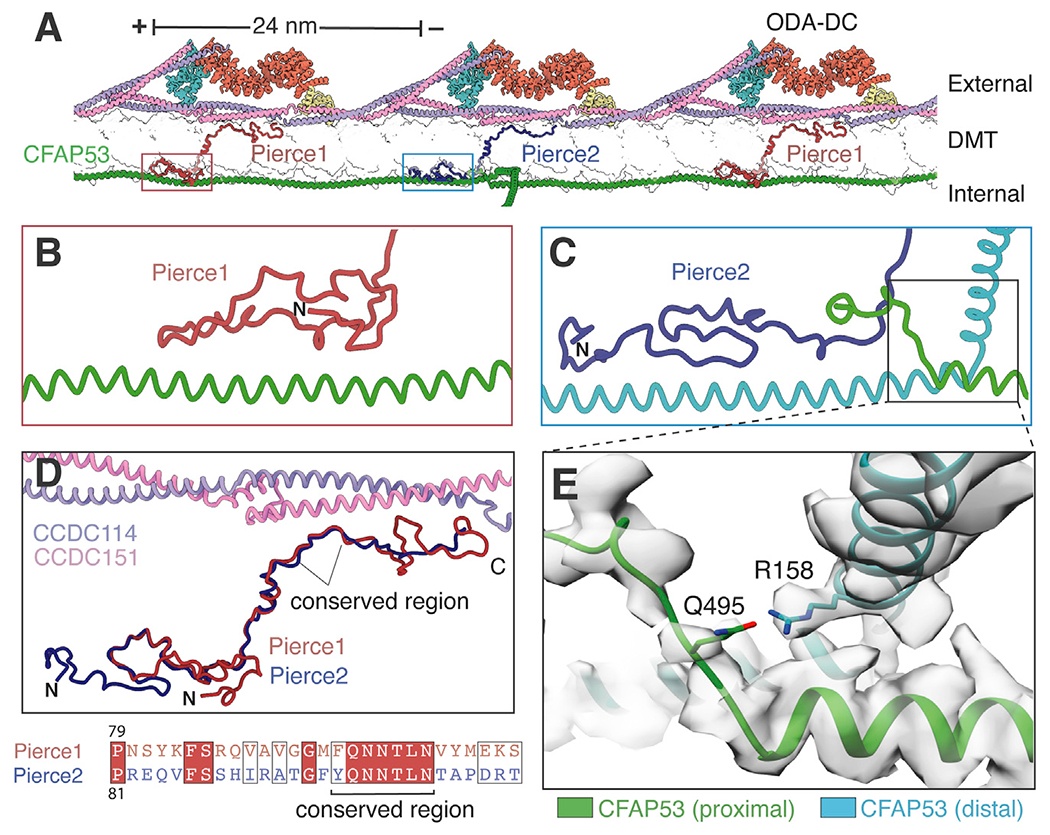
Pierce1 and Pierce2 link the ODA-DC to the MIP architecture (A) Atomic model showing Pierce1 and Pierce2 spanning the microtubule wall (shown in transparent surface representation) and linking the external ODA-DC to the filamentous MIP CFAP53. (B and C) Interaction of the Pierce1 (B) and Pierce2 (C) N termini with CFAP53. (D) Superposition of Pierce1 and Pierce2. A conserved central region interacts with the CCDC114/151 coiled coil. The sequence alignment of this conserved region is shown beneath. (E) Expanded view showing a potential hydrogen bond between two neighboring CFAP53 molecules involving R158 and Q495. Mutation of R158 to glycine has been implicated casually in dextrocardia (Noël et al., 2016). See also Video S1.

The ODA-DC is linked to the internal MIP structure through two paralogous MIPs (Pierce1 and C15orf65) (Figure 5; Video S1). These MIPs bind to distinct regions of CFAP53 through their N termini (Figures 5B and 5C) and protrude through the microtubule wall to interact with identical regions of neighboring copies of the CCDC114/151 coiled coil through conserved C-terminal regions (Figure 5D). Because the name “Pierce” is apt for proteins that pierce the microtubule wall, we renamed *C15orf65* and its mouse and zebrafish orthologs (*Ccpg1os* and *c18h15orf65*, respectively) *Pierce2.* Pierce1 and Pierce2 are small proteins that resemble the C terminus of *Chlamydomonas* FAP182 but lack the large N-terminal nucleotidyltransferase domain found in algae (Ma et al., 2019). In mammals, Pierce1 and Pierce2 are spaced 24 nm apart but bind the same CFAP53 protein (Figure 5A), leading us to speculate that they may function to link the 48-nm periodicity of the MIP architecture with the 24-nm periodicity of the ODA-DC. To test whether these paralogs are required for ciliary motility, we ablated their genes from two model organisms, zebrafish and mice, and examined whether their loss caused ciliopathy-like phenotypes, loss of ciliary motility, and/or loss of ODAs from axonemes.

### Zebrafish *pierce1; pierce2* double knockouts exhibit ciliary motility and laterality defects

**Figure 6.**
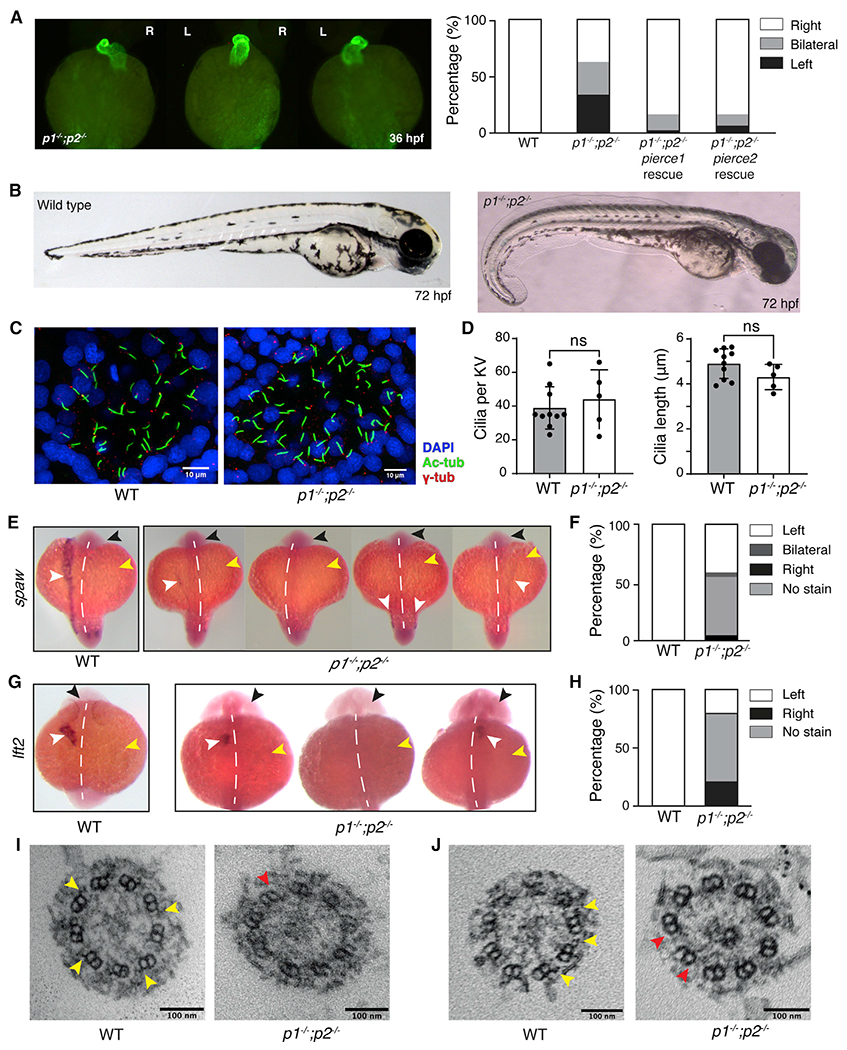
Laterality defects in *pierce1; pierce2* (*p1*^−/−^;*p2*^−/−^) double-mutant zebrafish embryos (A) Left: Immunofluorescence (IF) microscopy showing heart jogging directionality at 36 hpf. Images from left to right show the three heart jogging directions observed when imaged ventrally: rightward jogging (normal *situs*), bilateral jogging, and leftward jogging. Right: quantification of heart jogging directionality in WT (n = 61) and *p1*^−/−^;*p2*^−/−^ (n = 72) embryos and *p1*^−/−^;*p2*^−/−^ embryos following injection of *pierce1* (n = 43) or *pierce2* (n = 47) mRNA. (B) Bright-field images of WT (left) and *p1*^−/−^;*p2*^−/−^ (right) embryos at 72 hpf. (C) IF of KVs of WT (left) and *p1*^−/−^;*p2*^−/−^ (right) embryos using antibodies against acetylated tubulin (labeling axonemes), γ-tubulin (labeling basal bodies), and DAPI (labeling nuclei). (D) Quantification of cilium numberand length per KV in WT and *p1*^−/−^;*p2*^−/−^ embryos. Data are represented as mean ± SD (standard deviation). Unpaired t test was used to evaluate significance. (E) Whole-mount *in situ* hybridization for *spaw* on WT and *p1*^−/−^;*p2*^−/−^ embryos at the 18-somite stage. The black arrow indicates the head, the yellow arrow indicates the yolk, and the white arrow indicates the stain. The white dashed line indicates the embryonic midline. For *p1*^−/−^;*p2*^−/−^ embryos, expression on the left, no expression, bilateral and right expression, respectively, are depicted. (F) Quantification of *spaw* expression for WT (n = 36) and *p1*^−/−^;*p2*^−/−^ (n = 20) embryos. (G) Whole-mount *in situ* hybridization for *lft2* on WT and *p1*^−/−^;*p2*^−/−^ embryos at the 22-somite stage. Arrows and labeling are as in (E). (H) Quantification of *lft2* expression for WT (n = 62) and *p1*^−/−^;*p2*^−/−^ (n = 43) embryos. (I and J) Micrographs showing cross-sections from 9+0 (I) and 9+2 (J) KV cilia from WT and *p1*^−/−^;*p2*^−/−^ embryos. Yellow arrows indicate examples of ODAs, which are missing from *p1*^−/−^;*p2*^−/−^ embryos (red arrows). In *p1*^−/−^;*p2*^−/−^ embryos (n = 2), all 7 KV cilia examined lacked ODAs. See also Figure S5, Table S3, and Video S3.

First we generated zygotic single homozygous mutants of *pierce1* and *pierce2* in zebrafish using CRISPR gene editing (Figures S5A–S5E). Because there is maternal contribution of *pierce1* mRNA in zebrafish embryos (Figure S5F), we also generated maternally zygotic mutants by breeding the zygotic homozygous mutants. Neither the individual zygotic nor the maternally zygotic mutants displayed obvious phenotypic changes redolent of dysfunctional motile cilia, suggesting that Pierce1 and Pierce2 could be functionally redundant in zebrafish. We therefore generated maternally zygotic *pierce1*^−/−^; *pierce2*^−/−^ double knockouts (hereafter referred to as *p1*^−/−^;*p2*^−/−^). The double knockout embryos displayed heart defects 36 hours post fertilization (hpf), with approximately one third of embryos showing each of three phenotypes: rightward-jogged (normal), bilateral, and leftward-jogged hearts (Figure 6A). Notably, none of the embryos displayed complete looping of the heart tube in either direction. At 72 hpf, the mutant embryos displayed a mild curled-down phenotype at the distal end of their anterior-posterior axis (Figure 6B) that persisted throughout larval development. Consistent with this defect, adult *p1*^−/−^;*p2*^−/−^ fish displayed scoliotic spines of differing severities.

Because of the presence of a prominent heart laterality defect in the double mutants, we investigated cilia motility in Kupffer’s vesicle (KV), the LRO of teleost fish. At 14 hpf, KV contains 9+0 and 9+2 cilia. Although confocal imaging of double knockout embryo KVs (Figure 6C) showed no difference in cilium length or number relative to wild-type (WT) (Figure 6D), high-speed video microscopy revealed that KV cilia were almost completely immotile (Video S3). Interestingly, the motility of 9+2 cilia in pronephric ducts and nasal placodes was indistinguishable from that of the WT (Video S3), suggesting tissue-specific sensitivity to loss of Pierce1 and Pierce2. In zebrafish, proper axial development is dependent on cilium-driven flow of cerebrospinal fluid in the spinal canal (Zhang et al., 2018). Defective motility of spinal cilia is therefore the likely cause of axial curvature in the *p1*^−/−^*;p2*^−/−^ mutants (Figure 6B).

Given the paralysis of KV cilia in the *p1*^−/−^*;p2*^−/−^ mutant embryo, we investigated the possible downstream effect on the laterality markers *southpaw* (*spaw*), the zebrafish *Nodal* homolog, and *lefty2* (*lft2*), an inhibitor of Nodal signaling expressed in heart precursor cells. In WT zebrafish, expression of both genes is usually restricted to the left lateral plate mesoderm (LPM) (Figures 6E–6H). Strikingly, in *p1*^−/−^*;p2*^−/−^ embryos, we observed a severe reduction and loss of asymmetry of *spaw* expression (Figures 6E and 6F) as well as *lft2* expression (Figures 6G and 6H). *spaw* was either not expressed in the LPM or expressed weakly in the left or right LPM or bilaterally (Figures 6E and 6F). Likewise, *lft2* was expressed on the right, left, or not at all (Figures 6G and 6H), consistent with the three heart-jogging phenotypes described above.

Because *pierce1* and *pierce2* individual knockouts were phenotypically normal, we hypothesized that complementation of the double mutants with either gene should rescue the phenotypic defects. We therefore injected WT *pierce1* or *pierce2* sense mRNA into one-cell stage *p1*^−/−^*;p2*^−/−^ eggs. Examination of the resulting embryos at 36 hpf showed significant rescue in heart jogging direction (Figure 6A) and axial development. The rescue of heart laterality defects was confirmed to have arisen from restoration of KV ciliary motility (Videos S3C and S3D).

### *Pierce1/2* deficiency causes abnormal ciliary motility and *situs* defects in mice

**Figure 7.**
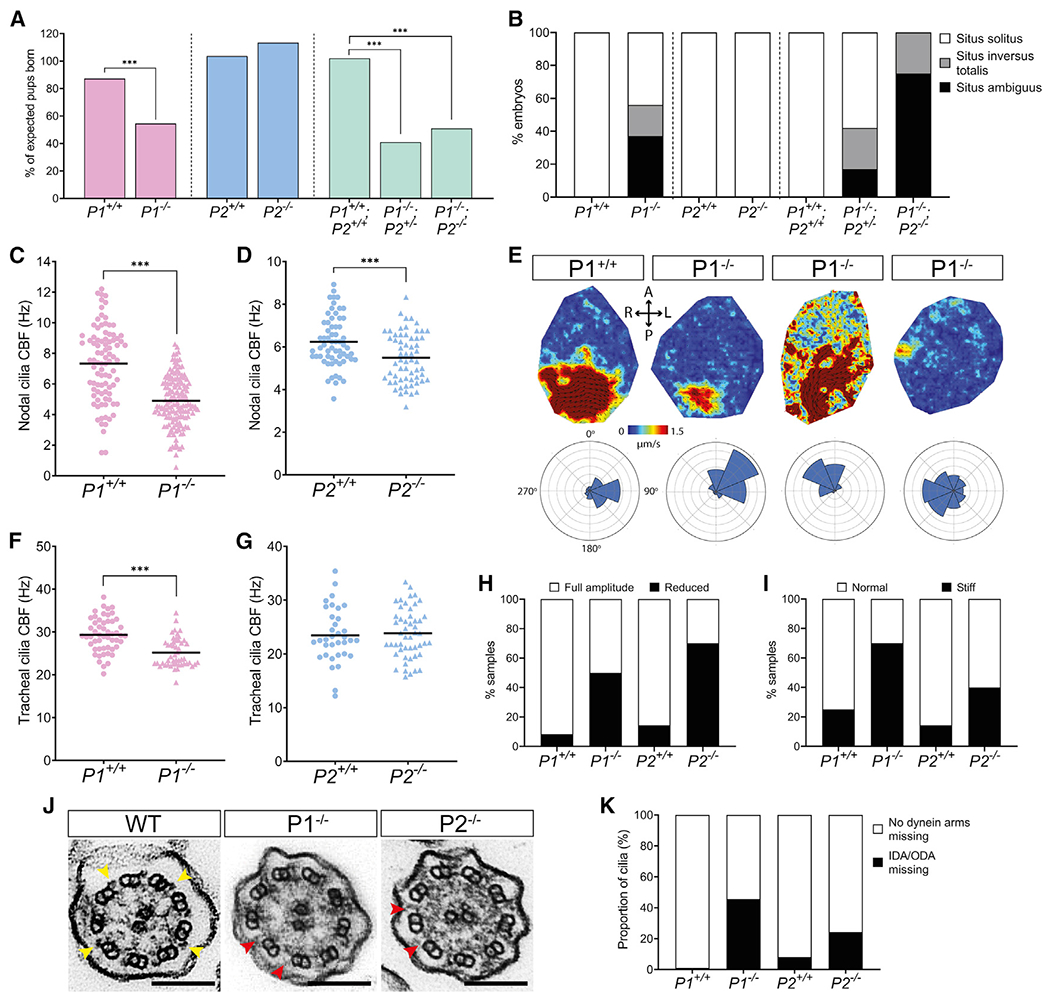
*Pierce1*-deficient mice have aberrant nodal and tracheal cilia motility (A) *Pierce1 (P1)*^−/−^ and *P1*^−/−^;*P2*^−/−^ double knockout mice have high embryonic lethality, whereas *P2*^−/−^ mice do not. All other genotypes were born at the expected frequencies. Chi-square analysis was used to evaluate significance. (B) *P1*^−/−^ and *P1*^−/−^;*P2*^−/−^ embryos have visceral organ *situs* defects. A bar chart shows the proportion of embryos displaying *situs solitus* (normal organ positioning), *situs inversus totalis* (total inversion to normal), and *situs ambiguus* (abnormal organ positioning) for each genotype at E13.5. *P1*^*+/+*^ (n = 16), *P1*^−/−^ (n = 16), *P2*^+/+^ (n = 9), *P2*^−/−^ (n = 10), *P1*^+/+^;*P2*^+/+^ (n = 7), *P1*^−/−^;*P2*^+/−^ (n = 12) and *P1*^−/−^;*P2*^−/−^ (n = 8) are shown. All other genotypes displayed only *situs solitus*. (C and D) Nodal cilia beat frequency (CBF) is reduced significantly in *P1*^−/−^ and *P2*^−/−^ embryos at E8.0. Average CBF is 7.3 Hz versus 4.9 Hz (*P1*^*+/+*^ versus *P1*^−/−^) and 5.9 Hz versus 5.1 Hz (*P2*^*+/+*^ versus *P2*^−/−^). Numbers of embryos analyzed are 19, 36, 9, and 8 for *P1*^*+/+*^, *P1*^−/−^, *P2*^+/+^, and *P2*^−/−^, respectively. CBFs of 5–10 cilia were quantified per node. ***p < 0.001 Student’s t test. Heterozygous genotypes did not show significant differences in their mean CBF. (E) Mean fluid velocity is reduced and/or directionality is abnormal in *P1*^−/−^ embryonic nodes at E8.0. Dark red refers to high velocity (1.5 μm/s), and dark blue refers to low velocity (0 μm/s). Localized directionality of flow is shown by black arrows. Anterior (A), posterior (P), left (L), and right (R) axes are annotated. Overall directionality of flow is depicted in rose plots in the bottom panel; vector direction is indicated in 8 directional segments, with the number of vectors indicated by the size of the segment. *P1*^*+/+*^ (n = 8) embryos display an organized, leftward nodal fluid flow, whereas *P1*^−/−^ (n = 8) embryos show a range of unusual phenotypes, including leftward flow (n = 2), disordered flow (n = 4), and weak flow with no overall directionality (n = 2) (panels from left to right). *P1*^*+*/−^ did not differ compared with WT embryos. (F and G) Tracheal CBF is reduced in *P1*^−/−^ but not *P2*^−/−^ mice. Average CBF is 29.4 Hz versus 25.2 Hz (*P1*^*+/+*^ versus *P1*^−/−^) and 23.4 Hz versus 23.8 Hz (*P2*^*+/+*^ versus *P2*^−/−^). 7–11 trachea were harvested for each genotype, with 5 ring sections assessed per trachea. ***p < 0.001 Student’s t test. *P2*^*+*/−^ mean CBF did not differ compared with WT embryos. (H and I) Tracheal cilia beat pattern is disrupted in *P1*^−/−^ and *P2*^−/−^ adult mice. Bar charts show the proportion of *P1*^*+/+*^ (n = 12), *P1*^−/−^ (n = 10), *P2*^*+/+*^ (n = 7), and *P2*^−/−^ (n = 10) trachea displaying cilia with a reduced beat amplitude (H) and a stiff waveform (I). P1^+/−^ and *P2*^*+*/−^ did not differ compared with the WT. (J) TEM of tracheal cilia cross-sections from WT, *P1*^−/−^, and *P2*^−/−^ mice. Yellow arrows on the WT image indicate ODAs, whereas red arrows in the *P1*^−/−^ and *P2*^−/−^ images indicate missing dynein arm(s). Scale bars, 100 nm. (K) Quantification of dynein arm defects observed in adult mouse tracheal cilia from *P1*^*+/+*^ (n = 3,387 cilia), *P1*^−/−^ (n = 4,597 cilia), *P2*^*+/+*^ (n = 3,604 cilia), and *P2*^−/−^ (n = 3, 540 cilia) genotypes. ~46% of *P1*^−/−^ and ~24% of *P2*^−/−^ tracheal cilia axonemes have missing dynein arms. Micrographs were assessed by three independent evaluators blind to genotype. See also Figure S6, Table S3, and Videos S4 and S5.

The observation that *p1*^−/−^*;p2*^−/−^ zebrafish exhibit abnormal laterality (Figure 6) prompted us to determine whether double-mutant mice exhibited similar ciliopathy-like phenotypes. Using CRISPR gene editing, we generated mice carrying a null allele of *Pierce2* (*P2*) and crossed them with mice carrying a LacZ-tagged null allele of *Pierce1* (*P1*) (Figures S6A–S6D). High levels of embryonic and pre-weaning lethality were evident in the double mutants (Figure 7A). The few mice that survived birth (n = 5) displayed hydrocephalus and laterality abnormalities, dying by 20 days of age. Strikingly, over half of embryonic day 13.5 (E13.5) *Pierce1*^−/−^ embryos examined displayed *situs* defects (Figure 7B; Figure S6D). Severe laterality abnormalities have also been reported previously for a gene-trapped *P1* null allele (Sung et al., 2016), although association of Pierce1 function with nodal cilia motility was not explored in that study. We hypothesized that the *situs* defects evident in *P1*^−/−^ mice resulted from defective ciliary motility in the embryonic node, the mammalian LRO analogous to the zebrafish KV. To assess nodal cilia motility, we imaged embryos at E8.0 blind to genotype (Video S4). The beat frequency of nodal cilia in *P1*^−/−^ embryos was reduced significantly compared with the WT (Figure 7C). *P2*^−/−^ embryos were less affected (Figure 7D). We also detected frequent abnormalities in the ciliary beat pattern in *P1*^−/−^ embryos. To determine how these changes affected nodal flow, we used particle image velocimetry (PIV), which utilizes ectopically applied fluorescent beads to assess the speed and directionality of flow over time (Figure 7E). As expected, WT and heterozygous embryos showed a strong and organized leftward nodal flow, consistent with published data (Nonaka et al., 1998; Shinohara et al., 2012). However, nodal flow was disrupted significantly in *P1*^−/−^ embryos, with loss and confused directionality of flow.

To determine the effect of impaired nodal flow in *P1*^−/−^ embryos, we analyzed the spatial expression of two key genes for left-right asymmetry, *Cerl2* and *Pitx2.* In WT mice, flow causes *Cerl2* mRNA to be downregulated on the left side of the node (Nakamura et al., 2012); subsequently, *Pitx2* is expressed exclusively in the left LPM (Ryan et al., 1998). For both genes, we observed less pronounced asymmetry in *P1*^−/−^ embryos than in WT and heterozygous embryos (Figure S6). These findings are consistent with loss of asymmetric expression of multiple node-associated genes in *P1*-null mice (Sung et al., 2016). Loss of asymmetry in left-right gene expression is well established to lead to laterality defects (Little and Norris, 2021), leading us to conclude that the *situs* defects of *P1*-deficient mice are caused by impaired ciliary motility and leftward nodal flow in the embryonic node.

The motility of tracheal cilia in adult mice was also affected by *P1* deficiency. Live imaging revealed a modestly reduced ciliary beat frequency (Figure 7F; Video S5) with a reduced amplitude of beating (Figure 7H) and an increased incidence of stiffly beating cilia (Figure 7I). The ciliary beat frequency in *P2*^−/−^ mice appeared to be unaffected (Figure 7G); however, effects similar to loss of *P1* upon amplitude and cilium beat stiffness were observed (Figures 7H and 7I; Video S5).

Because our structures suggested that the Pierce proteins could help attach ODAs to DMTs and link 24- and 48-nm periodicities (Figure 5), we examined the ultrastructure of KV cilia in WT and double-mutant zebrafish embryos and tracheal cilia in adult WT and *P1*^−/−^ and *P2*^−/−^ mice using transmission electron microscopy (TEM). The micrographs showed near-complete loss of ODAs from KV cilia (Figures 6I and 6J) and loss of dyneins from almost half of *P1*^−/−^ and a quarter of *P2*^−/−^ tracheal axonemes (Figures 7J and 7K). Thus, loss of axonemal dyneins explains the impaired motility of tracheal and LRO cilia in *Pierce1/2* mutants.

## DISCUSSION

### *De novo* identification of mammalian MIPs and luminal tektin filaments

Through this work, we defined 29 proteins, many of which were previously uncharacterized, as MIPs. Notably, we determined the structure, position, and higher-order organization of mammalian tektins 1–4. Tektins have been described as evolutionarily and structurally related to intermediate filaments based on their shared properties of high chemical stability, approximate 10-nm diameter made up of oligomerized filaments, and central rod domains with four predicted helices (Amos, 2008). However, the helix-turn-helix organization of the tektin fold identified here is incompatible with current models for assembly of intermediate filaments (Eldirany et al., 2021; Herrmann and Aebi, 2016) and their proposed coiled-coil architecture (Chernyatina et al., 2012; Nicolet et al., 2010). We therefore consider tektins and intermediate filaments to be structurally distinct classes of cytoskeletal filaments.

Tektins are apparent in cryo-ET maps of human tracheal cilia (Lin et al., 2014b) and mammalian sperm flagella (Leung et al., 2021). However, the variable number of tektin paralogs across eukaryotic lineages (Bastin and Schneider, 2019) and the absence of tektins from the A tubule of *Chlamydomonas* DMTs (Ma et al., 2019) suggests that tektins have species- or clade-specific architectures, binding sites, and compositions. These differences rule out tektins functioning as conserved molecular rulers for axonemal complexes (Pirner and Linck, 1994). Given the stability of tektins, it is more likely that they stabilize DMTs to withstand waveform-dependent mechanical forces, consistent with the appearance of structural defects in mouse sperm flagella in tektin 3 mutants (Roy et al., 2009).

Tektins have also been localized by immunofluorescence and immunoelectron microscopy to centrioles and basal bodies (Steffen and Linck, 1988; Stephens and Lemieux, 1998; Yanagisawa and Kamiya, 2004) and outside of axonemes in sperm flagella (lida et al., 2006; Murayama et al., 2008; Takiguchi et al., 2011). However, subtomogram averages of mammalian centrioles, basal bodies, and transition zones (Greenan et al., 2018; 2020) show no density consistent with tektin bundles (Figure S4B), suggesting that tektin bundles in respiratory cilia localize predominantly to the axoneme. Furthermore, the conserved 16-nm repeat of tektins implies strong coevolution with the 8-nm repeat of microtubules and that any role of tektins outside axonemes is secondary to their original function.

### Identification of diverse ODA docking mechanisms

Our work demonstrates the existence of at least two types of ODA-DCs: a trimeric ODA-DC found in algae (Takada et al., 2002; Walton et al., 2021) and ciliates (Kubo et al., 2021) and a pentameric ODA-DC found in mammals. Despite their differences in size and composition, there are several similarities. The periodicities of both types are enabled by cleft-occupying 24-nm coiled coils, both have a calcium-responsive subunit (DC3 in the trimeric complex [Casey et al., 2003] and Calaxin in the pentameric complex), and both interact with the tails of the dynein heavy chains. The two types of ODA-DCs may be an evolutionary response to binding ODAs with different numbers of motor domains. *Chlamydomonas* and *Tetrahymena* have triple-headed ODAs and a trimeric ODA-DC, whereas species with double-headed ODAs appear to have pentameric ODA-DCs, based on sequence analysis and visual assessment of subtomogram averages of axonemes (Figure S4A; Lin et al., 2014a; Yamaguchi et al., 2018). *Trypanosoma brucei* may be an outlier because it contains a double-headed ODA but lacks obvious orthologs of ARMC4 and TTC25 and has an axoneme with density inconsistent with the pentameric ODA-DC (Figure S4A; Imhof et al., 2019).

We speculate that the ODA-DC, as well as tethering dyneins to DMTs, may play an active role in regulating ciliary motility through two separate mechanisms. The first mechanism, based on the observation that curvature-induced lattice compression can reposition the ODA-DC (Figure 4), hypothesizes that asymmetric compressive forces within the beating axoneme alter the conformational state of ODA-DC and its associated dynein motor. We propose that higher curvature leads to more active dyneins based on cryo-ET studies of rapidly frozen sea urchin sperm flagella showing that active dyneins cluster in a bend-direction-dependent manner that correlates with compression of the A tubule (Lin and Nicastro, 2018). The second mechanism proposes that Calaxin acts as a conduit to relay changes in calcium concentration to directly modulate dynein behavior. This mechanism is consistent with the ability of sea urchin Calaxin to suppress ODA-driven microtubule sliding *in vitro* at high calcium concentrations (Mizuno et al., 2012).

### A mechanism for bridging 48- to 24-nm periodicity across the microtubule wall

Spatial coordination of the internal and external periodicities of microtubules is a universal feature of axonemes. We identify how two paralogs, Pierce1 and Pierce2, provide an ingenious solution to the problem of transitioning between periodicities across microtubule walls. Both paralogs make conserved interactions with the 24-nm ODA-DC through similar sequences in their C terminus but bind different sequences of the 48-nm filamentous MIP, CFAP53, through their diverged N termini (Figure 5). The observation that CFAP53 mediates the crosstalk between MIPs and the ODA-DC explains why *Cfap53*-deficient zebrafish and mice have cilia motility defects because of lack of ODAs (Ide et al., 2020; Narasimhan et al., 2015) and why mutations in human *CFAP53* cause laterality abnormalities (Narasimhan et al., 2015; Noël et al., 2016; Perles et al., 2012). Using gene editing, we show that loss of *pierce1* and *pierce2* in zebrafish and mice affects rotating cilia more than planar beating cilia, which is consistent with previous studies showing that MIP defects mainly cause laterality abnormalities (Table S3). An explanation for this phenomenon is that the forces associated with rotation and the absence of auxiliary structures in 9+0 cilia make ODA loss more likely in rotating than beating cilia. Alternatively, there may be differences at the ODA docking site that remain to be discovered.

We also observed that Pierce1 and Pierce2 appear to be redundant in zebrafish but not mice. One possibility is that a single paralog bound every 48 nm is sufficient to generate 24- 48-nm periodicity in zebrafish but not mice because of species-specific differences in the ODA/ODA-DC. If these complexes have a strong propensity to self-assemble and remain assembled, then a single Pierce paralog may be sufficient. Alternatively, although Pierce1 and Pierce2 bind different regions of CFAP53, they bind identical regions of tubulin and CCDC114/151 (Figure 5). It is therefore possible that one Pierce paralog could partially occupy the binding site of the other paralog in its absence. If this compensatory mechanism occurred in zebrafish but not mice, then it could explain the differences in redundancy. Furthermore, if mouse Pierce1 could partially compensate for Pierce2 but not vice versa, then it could explain the more severe phenotype of the *P1* compared with the *P2* knockout mouse.

### A structural framework to understand ciliopathies

Thirteen proteins identified in this study are associated with human ciliopathies, and more have ciliopathy-like phenotypes in mouse and zebrafish mutants (Table S3). Our structures therefore provide a high-confidence candidate list to improve the diagnosis of PCD, for which ~25% of cases have no known genetic cause (Fassad et al., 2020). The structural information also provides a framework to interrogate the roles of individual axonemal proteins and their contribution to ciliary motility and ciliopathies, as we demonstrate for Pierce1 and Pierce2. In addition, our structure provides a reference to rationalize ciliopathy-causing mutations at the amino acid level, determine potential disease mechanisms, and prioritize variants of unknown significance. For example, our structure shows that a single residue substitution in CFAP53 (R158G) that has been implicated causally in dextrocardia (Noël et al., 2016), maps to the interface between adjacent CFAP53 molecules and could have a potentially destabilizing effect (Figure 5E).

### Limitations of the study

In our structure, some small sections of density remain unassigned because of insufficient resolution, and not all residues or side chains of the identified proteins can be resolved (Methods S1). Although our structure identifies the MIP repertoire of the bovine respiratory DMT, the repertoire may differ in other ciliated cell types. Of the identified MIPs, we focused on Pierce1 and Pierce2. Further work will be required to elucidate the roles of other MIPs, for which our structure will help guide the design of genetic experiments. Our functional characterization demonstrates that the *Pierce1* and *Pierce2* genes are important for ciliary motility and that their genetic loss causes ciliopathy-like phenotypes. However, full characterization of double-mutant mice was not possible because few survived birth or to the stage when tracheal cilia could be analyzed. Although the reason why *Pierce1* loss leads to a more severe phenotype in mice than zebrafish and why ciliary motility is affected differently in different tissues requires further investigation, our data nevertheless reinforce the existence of considerable diversity in the different cilium types within the vertebrate body.

## STAR★METHODS

### RESOURCE AVAILABILITY

#### Lead contact

Further information and requests for resources and reagents should be directed to and will be fulfilled by the Lead Contact, Alan Brown (alan_brown@hms.harvard.edu).

#### Materials availability

Zebrafish lines generated by the authors will be distributed without restriction upon request. The mouse *1700007K13Rik*^tm2b(EUCOMM)Wtsi^ and *Ccpg1os*^em1(IMPC)H^ alleles are available through the International Mouse Phenotyping Consortium (IMPC; https://www.mousephenotype.org/). Plasmids generated in this study will be distributed without restriction on request.

#### Data and code availability

The cryo-EM map of the outer dynein arm (ODA) core from bovine tracheal cilia and the composite cryo-EM map of the 48-nm repeat of the bovine DMT have been deposited in the Electron Microscopy Data Bank with accession codes EMD-24663 and EMD-24664, respectively. The consensus map, mask, and local refined maps have been deposited as additional files. The atomic model of the 48-nm repeat of the bovine DMT has been deposited in the Protein Data Bank with the accession code 7RRO.

This paper does not report original code.

Any additional information required to reanalyze the data reported in this paper is available from the lead contact upon request.

### EXPERIMENTAL MODEL AND SUBJECT DETAILS

#### Bacterial strains

*Escherichia coli* DH5α cells (Thermo Fisher Scientific and New England Biolabs) were used for the propagation of all vectors and plasmids. Bacterial colonies were cultured on LB agar plates or in liquid LB medium supplemented with the appropriate antibiotic for selection at a concentration of 100 μg/ml. LB agar plates and liquid cultures were incubated for 14-16 hours at 37°C prior to isolation of vector or plasmid DNA. Transformations were carried out following an established standard protocol.

#### Zebrafish husbandry, strains, and mutagenesis

Zebrafish strains used in this study were maintained at the Institute of Molecular and Cell Biology (IMCB, Singapore) zebrafish facility following routine husbandry procedure. The facility has a controlled temperature of 28.5°C and operates a 14-hr light and 10-hr dark light cycle. All experiments with the zebrafish were conducted with approval of the Singapore National Advisory Committee on Laboratory Animal Research. The zebrafish strains used in the study are listed in the Key resources table. All experiments were carried out on zebrafish embryos and larvae between 10 somites and 72 hpf stages. There was no sex bias and paired matings were set up using adult zebrafish between 4 and 8 months of age.

#### Mouse husbandry, stains, and mutagenesis

Ethical approval for all mouse work was obtained from the UK Home Office and experiments were carried out in accordance with the Medical Research Council (MRC) Harwell Ethics Committee. All mouse colonies were maintained in a pathogen-free environment at the Mary Lyon Centre, MRC Harwell Institute on a C57BL/6N background strain. The *1700007K13Rik*^tm2b(EUCOMM)Wtsi^ allele (*Pierce1*^tm2b)^ was created at the Wellcome Sanger Institute as part of the European Conditional Mouse Mutagenesis Program (EUCOMM). The *Ccpg1os*^em1(IMPC)H^ allele (*Pierce2*^DEL^) was created at the Mary Lyon Centre as part of the International Mouse Phenotyping Consortium (IMPC). Mice were housed in groups of 2-5 with controlled temperature (21 ± 2°C) and humidity (55 ± 10%) in a 12-hour light/dark cycle. Mice had free access to water and were fed *ad libitum* on a commercial diet (Special Diet Services, UK). Mice were sacrificed either by cervical dislocation or overdose of anesthetic. The mouse embryos analyzed were a random mixture of males and females; their sex was not determined. Adult mouse cohorts included equal numbers of males and females; no sex-based phenotypic differences were detected.

### METHOD DETAILS

#### Isolation of bovine tracheal cilia

The protocol for isolating bovine tracheal cilia was modified from (Anderson and Hein, 1976; Hastie, 1995; Hastie et al., 1986). Fresh bovine tracheae were collected from Adam’s Farm (Athol, MA) and stored in PBS (137 mM NaCl, 2.7 mM KCl, 10 mM Na_2_HPO_4_, 1.8 mM KH_2_PO4, pH 7.4) on ice for the drive back to the lab (approximately 90 min). The following extraction and purification steps were carried out at 4°C. The tracheae were washed with PBS and excess tissue was removed. A nylon brush was carefully inserted into the trachea to brush the epithelium lightly. The brush was washed with about 100 mL extraction buffer (20 mM Tris, pH 7.4, 50 mM NaCl, 1 mM Ethylenediaminetetraacetic acid (EDTA), 7 mM β-mercaptoethanol, 10 mM CaCl_2_, 250 mM sucrose, 0.1% 3-[(3-Cholamidopropyl) dimethylammonio]-1-propanesulfonate (CHAPS) (w/v)), which was subsequently filled into the trachea. Both ends of the flesh tube were sealed by parafilm and rubber bands, and the trachea filled with extraction buffer was shaken vigorously for about 2 min. The buffer was collected, and the trachea was rinsed with another 100 mL extraction buffer without CHAPS. The combined buffer samples (final concentration of CHAPS: 0.05%) were passed through a sieve to separate any residual tissue. The flow through was filled into 1 L centrifugation tubes and centrifuged at 2,000 × g for 2 min. The supernatant was carefully transferred to 175 mL conical centrifugation tubes and centrifuged at 12,000 × g for 30 min. The cilia containing pellet was resuspended in RB buffer (30 mM HEPES, pH 7.4, 5 mM MgCl_2_, 1 mM 1,4-dithiothreitol (DTT), 0.5 mM EDTA, 50 mM KCl, protease inhibitor (Roche)). Several rounds of low speed (2,000 × g) and high speed (13,300 × g) centrifugation were performed to clean up the cilia. The final cilia pellet was resuspended in RB buffer. The sample was analyzed by negative-stain electron microscopy for cilia concentration, integrity, and purity. The sample was flash-frozen in liquid nitrogen and stored at −80°C.

#### Preparation of bovine DMTs

The purified cilia were demembranized by adding NP-40 detergent (Thermo Fisher Scientific) to a final concentration of 0.5% and incubated at 4°C for 30 min. The sample was centrifuged at 12,000 × g for 20 min. The supernatant was removed and the pellet was resuspended in RB buffer to an A280 concentration of 0.5-2.1 mM ATP was added to the sample and incubated at room temperature for 20 min. If any pellet remained after incubation, ATP up to a concentration of 2.5 mM was added and the sample was incubated again at room temperature for another 10-20 min. After incubation, the sample was centrifuged at 6,000 × g for 20 min and the pellet was resuspended in RB buffer to an A280 concentration of ~10 for cryo-grid preparation.

#### Purification of tektin filaments

Tektin filaments were purified following a published protocol (Pirner and Linck, 1994). Briefly, purified cilia were demembranized by adding CHAPS detergent to a final concentration of 2% and incubated at 4°C for 30 min, followed by a centrifugation at 6,000 × g for 10 min. The pellet was resuspended in tektin buffer (0.5% Sarkosyl, 50 mM Tris, pH 8, 1 mM EDTA, 1 mM DTT). Urea was added to the sample to a final concentration of 2 M. The sample was incubated at room temperature for 1 hour before diluting the urea concentration to 0.8 M. The sample was then subjected to ultracentrifugation using a SW50.1 rotor at 100,000 × g for 90 min at 16°C. The supernatant was discarded, and the pellet was resuspended in 20 mM HEPES, pH 7.4, 5 mM MgSO4, 1 mM DTT, 1 mM EGTA, 100 mM KCl containing 1× ProteaseArrest protease inhibitors (G-Biosciences). The sample was denatured with SDS-loading buffer and loaded onto a 4%–20% precast polyacrylamide gel (Bio-Rad). The gel was silver stained and the bands between 10 kDa to 40 kDa were cut and sent for mass-spectrometry analysis. Additionally, another gel was run for 3 min and stained with Coomassie blue. The bands containing the whole sample was cut and sent for mass-spectrometry analysis.

#### Mass-spectrometry analysis

The purified bovine DMTs and tektin samples were sent for mass spectrometry analysis at the Taplin Mass Spectrometry Facility at Harvard Medical School. The bovine DMT was provided in solution, and the tektin samples as bands excised from SDS-PAGE gels. The gel pieces were washed and dehydrated with acetonitrile. After 10 min, the acetonitrile was removed, and the gel pieces dried using a SpeedVac vacuum concentrator (Thermo Fisher Scientific). The gel pieces were then rehydrated with 50 mM ammonium bicarbonate solution containing 12.5 ng/μl trypsin (Promega). After 45 min at 4°C, the trypsin solution was replaced with 50 mM ammonium bicarbonate solution. Samples were then placed at 37°C overnight. Peptides were later extracted by removing the ammonium bicarbonate solution, followed by one wash with a solution containing 50% acetonitrile and 1% formic acid. The extracts were then dried in a SpeedVac (~1 hr) and stored for use at 4°C. On the day of analysis, the samples were reconstituted in 5-10 ~l of solvent A (2.5% acetonitrile, 0.1% formic acid) and loaded onto a pre-equilibrated reverse-phase capillary column (100 ~m inner diameter × ~30 cm length) containing 2.6 μm C18 spherical silica beads using a Famos auto sampler (LC Packings). A gradient was formed, and peptides were eluted with increasing concentrations of solvent B (97.5% acetonitrile, 0.1% formic acid). As peptides eluted they were subjected to electrospray ionization and then entered into an LTQ Orbitrap Velos Pro ion-trap mass spectrometer (Thermo Fisher Scientific). Peptides were detected, isolated, and fragmented to produce a tandem mass spectrum of specific fragment ions for each peptide. Protein identity was determined from the acquired fragmentation pattern using Sequest (Thermo Fisher Scientific).

The data were filtered to between a one and two percent peptide false discovery rate. DMTs in solution were treated similarly but were desalted after digestion. The results of the analysis are provided in Table S1.

#### Negative-stain electron microscopy

A 4 μL aliquot of bovine DMT sample at an A280 reading of ~2 was applied onto a glow discharged continuous carbon grid (Electron Microscopy Sciences). After one minute of adsorption, the grid was blotted with filter paper to remove the excess sample, immediately washed twice with 4 μL of 1.5% uranyl formate solution and incubated with 4 μL of 1.5% uranyl formate solution for an additional one minute. The grid was then further blotted with filter paper to remove the uranyl formate solution, air-dried at room temperature, and examined with CM10 electron microscope (Phillips) or Tecnai T12 electron microscope (Thermo Fisher Scientific). The CM10 is operated at 100 kV acceleration voltage with a tungsten filament and is equipped with a Gatan UltraScan 894 (2k × 2k) CCD camera. The T12 is operated at 120 kV acceleration voltage with an LaB6 filament and is equipped with a Gatan UltraScan 895 (4k × 4k) CCD.

#### Cryo-EM data collection

For cryo-EM analysis, 3 μL of bovine DMT sample with an absorbance reading at 280 nm of ~10 was applied onto glow discharged C-flat holy carbon grids (R1.2/1.3, 400 mesh copper, Electron Microscopy Sciences) or Quantifoil holy carbon grids (R1.2/1.3, 400 mesh gold, or R2/2, 400 mesh copper, Quantifoil Micro Tools). The grids were blotted for 9 to 11 s with a blot force of 16 in 100% humility before being plunged into liquid ethane cooled by liquid nitrogen by using a Vitrobot Mark IV (Thermo Fisher Scientific) at the Harvard Cryo-EM Center for Structural Biology. The grids were screened for good ice conditions with a Tecnai F20 microscope (Thermo Fisher Scientific) operating at 200 kV acceleration voltage with a FEG electron source and equipped with a K2 Summit direct electron detector (Gatan).

Images were acquired on Titan Krios I at the Harvard Cryo-EM Center for Structural Biology equipped with a BioQuantum K3 Imaging Filter (slit width 25 eV) and a K3 direct electron detector (Gatan) and operating at an acceleration voltage of 300 kV. Images were recorded at a defocus range of −1 μm to −2.5 μm with a nominal magnification of 81,000×, resulting in a pixel size of 1.09 A. Each image was dose-fractionated into 47 movie frames with a total exposure time of 2.8 s, resulting in a total dose of ~60 electrons per A^2^. SerialEM was used for data collection (Schorb et al., 2019). The images were acquired from five independent data collection sessions.

#### Image processing

All image processing was performed using RELION 3.1 (Zivanov et al., 2018) unless otherwise stated. A total of 33,755 movie stacks were motion corrected and electron-dose weighted using MotionCor2 (Zheng et al., 2017). A representative micrograph is provided in Figure S1A. Parameters of the contrast transfer function (CTF) were estimated from the motion-corrected micrographs using CTFFIND4 (Rohou and Grigorieff, 2015). 14,726 micrographs were selected for further processing based on visual inspection of the micrographs and their corresponding power spectra. Micrographs that lacked microtubules or had high drift were excluded. To pick particles, start and end points for the DMTs were manually selected. Both straight and curved microtubules were picked. Helical segmented particles were extracted with a helical rise of 8.2 nm and the number of asymmetrical units set to one. Particles were extracted in 672-pixel boxes and downscaled to 336-pixel boxes to accelerate computation. A round of two-dimensional classification served to verify data quality (Figure S1A). No particles were excluded at this stage. In total, 1,267,170 particles were extracted and subjected to three-dimensional (3D) refinement using the map of the *Chlamydomonas* DMT (EMDB: EMD-20631) (Ma et al., 2019) low-pass filtered to 15 Å as a reference. After refinement, the 8-nm particles were subjected to 3D classification to exclude particles of singlet microtubules and broken DMTs. 697,059 particles were retained and re-extracted without downscaling in 672-pixel boxes. These particles were subjected to 3D refinement, CTF refinement, Bayesian polishing, and another round of refinement. This particle set was used to determine maps of the 48-nm internal repeat and the external 24-nm repeat of the ODA-DC and ODA as described below. An overview of the processing strategy is provided in Figure S1 with detailed flow diagrams given in Methods S1.

#### Determination of a reconstruction of the internal 48-nm repeat

To obtain a reconstruction of the internal 48-nm repeat, we performed two rounds of classification: first to obtain the 16-nm repeat using a cylindric mask focused on the inner junction region of the B tubule and then to obtain the 48-nm repeat using a cylindric mask focused on the seam of the A tubule. This two-step process yielded better results than going directly from the 8-nm to the 48-nm repeat. The final 48-nm map is generated from 80,503 particles.

Density for the tektin bundle at the ribbon of the A tubule was less well-defined than other regions, indicating heterogeneity. We therefore performed focused classification using a cylindric mask over the bundle revealing classes corresponding to 0, 4- and 8-tektin filaments. Classes containing 4- or 8-tektin bundles were selected and independently refined. These maps were used to build atomic models of the tektin filaments.

To improve local map quality of the 48-nm repeat map, we performed a series of local refinements. First, we used 10 overlapping cylindric masks to divide the DMT into several subregions, each containing 2 or 3 protofilaments. We then used shorter cylindric masks to divide each subregion into three longitudinal sections. Using these methods, the resolution within each mask was improved. Additionally, we noticed that the density for protofilaments B02-B05 was poorly resolved. We therefore combined cylindric masks for subregions 7-9 and performed three-dimensional classification to isolate only those particles with well-defined density. The locally refined maps were used for model building.

#### Determination of a reconstruction of the external 96-nm repeat

To generate a map of the 96-nm external repeat, we performed a round of classification with a cylindric mask applied to the exterior of protofilaments A02 and A03, which is the binding site for many axonemal complexes which have 96-nm periodicities. The classification separates the two halves of the 96-nm repeat, one with radial spokes 1 and 2 (RS1 and RS2) and the other with RS3 and the nexin-dynein regulatory complex (N-DRC). Fitting our atomic model of the 48-nm repeat into both halves of the 96-nm repeat confirms the periodicity of the interior of the bovine DMT as 48 nm.

#### Determination of a reconstruction of the external 24-nm repeat

To generate a map of the 24-nm external repeat, we used a cylindric mask to classify density apparent on protofilaments A07-A08 of the 8-nm repeat map, corresponding to the ODA-DC. Classification identified all three different possible registers of the ODA-DC. We selected each class that showed good density for the ODA-DC and reboxed them with a smaller box size of 288 pixels to focus on the ODA-DC and shifted the density to the center of the box. We combined the particles and excluded duplicates, yielding 192,946 ODA-DC particles. We then performed three-dimensional classification on the DMT and the ODA-DC to further clean up the particles, generating 63,865 particles with well-resolved ODA-DC density. The particles were subjected to multi-body refinement leading to a 3.6 Å resolution map of the ODA-DC-bound microtubule and a 4.5 Å resolution map of the distal ODA-DC.

The 24-nm map was also used as a starting point to improve the 48-nm map of the luminal region beneath the ODA-DC. This region includes MIPs CFAP53, MNS1, Pierce1 and Pierce2. The 24-nm map was classified into four classes using a shaped mask that covered these MIPs. This identified two major classes: one centered on Pierce1 and one centered on Pierce2. Refinement of these two classes resulted in maps at 3.9 Å and 4.0 Å resolution, respectively. These maps were a qualitative improvement on maps generated starting from the 48-nm particles (Methods S1).

#### Determination of a reconstruction of the mammalian ODA

Particles with ODAs were identified using a strategy starting with the 48-nm repeat. Within the repeat, 1 copy and 2 partial copies of the ODA-DC were present. We reboxed each of these using a box size of 488 pixels and shifted them to a common center. After excluding duplicates, the particles were refined and reclassified to identify those with bound ODAs, followed by additional rounds of classification, refinement, and Bayesian polishing. Multi-body refinement of the remaining 8,755 particles was used to improve the map quality of the ODA. Refinement of the core of the ODA (excluding the motor domains but including the tail domains of the heavy chains and their associated light and intermediate chains) resulted in a 8 Å reconstruction.

The Fourier shell correlation (FSC) = 0.143 criterion (Rosenthal and Henderson, 2003) was used to calculate resolutions from independent half maps. Maps were postprocessed using phenix.auto_sharpen (Terwilliger et al., 2018) for visualization and deposition. DeepEMhancer, a neural network-based postprocessing approach, was used to sharpen the maps to guide model building (Sánchez-García et al., 2020).

#### Generation of composite maps

To generate a composite map for model building, refinement and deposition, locally refined maps were aligned using the *fit in map* command in Chimera (Pettersen et al., 2004) by maximizing the overlapped density and merged using the *vop maximum* command in Chimera. The individual maps that form the composite map of the bovine DMT are shown in Methods S1. The half maps of the corresponding local refined fragments were also merged and used for overall FSC calculation (Figure S1B) and local resolution estimation in RELION 3.1 (Figure S1D).

#### Model building

Model building was performed in Coot v0.9-pre or v0.9.4.1 (Brown et al., 2015). Interpretation of the bovine DMT map started with fitting of the atomic model of the *Chlamydomonas* DMT (PDB 6U42) (Ma et al., 2019). α- and β-tubulin isoforms were distinguished based on sidechain density. The most abundant isoforms identified by mass spectrometry (three for α-tubulin, TUBA1D, TUBA1B, and TUBA4A, and six for β-tubulin, TUBB1, TUBB2B, TUBB3, TUBB4B, TUBB5, and TUBB6) were aligned and the sidechain density inspected where the residues showed greatest variability. Particular attention was paid to locations where different isoform sidechains could be easily differentiated. For example, the density for β-tubulin at position 57 is most consistent with the glycine residue of TUBB4B rather than the bulkier sidechains of TUBB1, TUBB2B, TUBB3 and TUBB6 (lysine, asparagine, histidine and glutamine, respectively). Using this information, the α-tubulin isoform was assigned to TUBA1D based on sidechains of I16, G57, G59, H61,T334 and the β-tubulin isoform was assigned to TUBB4B based on sidechains of H37, N48, G57, V170, A365. These isoforms are consistent with single-cell RNA-sequencing showing their upregulation in ciliated airway cells compared with non-ciliated neighboring cells (Hawkins et al., 2021). However, we cannot exclude the possibility that other tubulin isoforms are incorporated into DMTs as minority species.

*Chlamydomonas* MIPs clearly lacking density in the bovine DMT were deleted from the atomic model. These were FAP34 (RIB30), FAP68, FAP85, FAP90, FAP112, FAP115, FAP129, FAP166, FAP22, FAP252, FAP273, FAP306 (RIB21), and FAP363. The remaining 22 MIPs were considered to have *Bos taurus* orthologs. We used the sequences of the *Chlamydomonas* MIPs to identify bovine orthologs from UniProt (UniProt Consortium, 2021) or the NCBI protein database (Sayers et al., 2021). The atomic models of the *Chlamydomonas* MIPs were mutated to match the sequence of the bovine proteins and loops and extensions were rebuilt. Sidechain density was used to distinguish between paralogs and isoforms. Additional paralogs were identified for FAP182 (Pierce1 and Pierce2) and RIB72 (EFHC1 and EFHC2). We also identified an additional copy of CFAP161. MIPs present in the bovine DMT but absent from *Chlamydomonas* (Tektins 1-4, TEKTIP1 (C19orf71), FAM166B and EFCAB6) were identified by *de novo* sequence assignment. Homology models of EFCAB6 were generated using SWISS-MODEL (Waterhouse et al., 2018) and TrRosetta (Yang et al., 2020) and were used to guide model building. Candidates for the additional MIPs were obtained from mass spectrometry analysis of the bovine DMT and extracted tektin samples (both Table S1) and the published proteome of human airway cilia (Blackburn et al., 2017).

ARMC4, CCDC114, CCDC151, and TTC25 were identified as components of the ODA-DC based on prior knowledge (Hjeij et al., 2013; 2014; Onoufriadis et al., 2013; Wallmeier et al., 2016) and sidechain density. The fifth component of the ODA-DC, Calaxin (EFCAB1), was identified by fold recognition using the MOLREP-BALBES pipeline (Brown et al., 2015). This pipeline identified a domain of two EF-hands (PDB 2OBH) as being the best fit with a contrast score of 4.2. We then searched our list of candidates from mass spectrometry (Table S1) for proteins with EF-hand motifs. Calaxin was the most likely solution given the colocalization of *Ciona* calaxin with ODAs (Mizuno et al., 2009). A homology model of bovine Calaxin was built using SWISS-MODEL and docked as a rigid body into the density using Coot. Following guidance from the HGNC, the five subunits of the mammalian ODA-DC are renamed outer dynein arm docking complex subunits ODAD1-5 (Methods S1).

The double-headed axonemal dynein of the ODA was interpreted by fitting the atomic model of the *Chlamydomonas* triple-headed axonemal dynein into the density (PDB 7KZM) (Walton et al., 2021) and deleting additional subunits not found in *Bos taurus* (α-HC and LC4).

#### Model refinement

Atomic models of individual subunits were refined during model building using real-space refinement in Coot with torsion, planar peptide, trans peptide and Ramachandran restraints applied (Brown et al., 2015). After model building, the subunits were combined into a single PDB file. The atomic model was then refined into the composite map using Phenix.real_space_refine v1.18.2-3874 (Afonine et al., 2018). Secondary structure, Ramachandran and rotamer restraints were applied during refinement. Rotamer restraints target was set to fix outliers and weighting of nonbonded restraints was set 1000. A round of manual model correction in Coot was performed between rounds of real-space refinement in Phenix. The final refinement was performed for three macro cycles with strategies of *minimization_global* and *local_grid_search.* The quality of the refined model was analyzed by MolProbity integrated in Phenix (Chen et al., 2010), with statistics reported in Table S2.

#### Structure analysis and bioinformatics

A curated list of 439 tektin protein sequences was obtained from a previous phylogenetic study (Bastin and Schneider, 2019). This list includes sequences from 111 species representing 24 metazoan phyla and *Cryptophyta, Chlorophyta* and *Choanoflagellata.* Of the 439 sequences, 249 were identified that aligned with the bovine Tektin 1 L2 loop upon protein basic local alignment search tool (BLAST) with an E-value threshold of 0.0001 and with a single well-defined L2 loop. The sequences were shifted to align their L2 loops with that of the longest tektin (*Clonorchis sinensis* Tektin 1). Each residue’s predicted secondary structure was plotted (Figure S3B). Secondary structure predictions were performed using PSIPRED (Jones, 1999). Evolutionary coupling (EC) analysis was performed using the EV-couplings V2 server (Hopf et al., 2019; Figure S3C). Sequences homologous to bovine Tektin 1 (UniProt ID Q32KZ9) were obtained using a search of the Uniprot90 database (UniProt Consortium, 2021). Intramolecular contacts for Tektin 1 were determined using CMView (Vehlow et al., 2011) with an 8 Å distance cutoff. Intermolecular contacts were determined using CONTACT from the CCP4 suite (Winn et al., 2011) with a 7 Å distance cutoff.

#### End-point PCR

RNA was isolated from zebrafish embryos with Trizol (Ambion Life Technologies, # 15596018). Reverse transcription (RT) PCR was carried out using one-step RT-PCR kit (QIAGEN, #210212). 500 ng of total RNA from each experimental group was used to synthesize the first strand cDNA. End-point PCRs on the zebrafish *pierce1* and *actin-b1* genes were performed on the cDNA templates. The PCR products were resolved on agarose gels and imaged using a gel imaging system (Bio-Rad). All primers are listed in Table S4.

#### CRISPR single guide RNA (sgRNA) design and synthesis

sgRNAs for the *pierce1* and *pierce2* genes were designed using the web tool CHOPCHOP (Montague et al., 2014). Target sites for the sgRNA were designed by seeking sequences corresponding to sequence GGN_18_NGG in the DNA. BLAST was used to identify off-target sites of the sgRNAs. sgRNAs with off-target sequences with no mismatches in the last 15 nt including the NGG PAM were discarded. The sgRNA templates were synthesized with Phusion High-Fidelity DNA polymerase (NEB, M0530S). Two primers were used: one forward primer with the T7 polymerase promoter and gene target sequence, and one reverse primer containing the remaining gRNA sequence. The sgRNAs were transcribed from the templates using the MEGAshortscript T7 Transcription Kit (Ambion, AM1354).

#### Cas9 and sgRNAs microinjection

A mixture of 800 ng of the Cas9 protein (Toolgen, Cat #TGEN_CP1) and 500 ng of sgRNA were incubated at 37°C for 15 min. 1 nL of this mixture was injected into the animal pole of one-cell stage embryos.

#### PCR analysis to identify mutants

Genomic DNA was extracted from embryos and fins from adult fish using the alkaline lysis method of 50 mM NaOH incubated at 95°C for 30 minutes, followed by neutralization with 40 mM Tris-HCl (Wilkinson et al., 2013). For embryonic genomic DNA extraction, 8-10 single embryos at 2 days old were used. To identify the mutations, primers were designed to bind upstream and downstream of the expected double-stranded breaks in the targeted exon. The PCR-amplified genomic DNA region from the mutants and WTs were cloned into a pCR II-TOPO vector and sequenced to confirm the mutation (Figure S5).

#### Morphological phenotype analysis of zebrafish embryos

Phenotypes were scored in live WT, *pierce1, pierce2* and the double knockout mutant embryos using a stereomicroscope. Phenotypes were analyzed in embryos at different stages. Otolith counts were performed at 20–22 hpf. Curved body axis and hydrocephaly were determined at 48 and 72 hpf. Kidney cysts and edema were scored at 4–5 days post fertilization (dpf). For left–right asymmetry, immunofluorescence microscopy was carried out at 36 hpf with anti-A4.1025 antibody (Developmental Studies Hybridoma Bank) to visualize heart jogging. Heart jogging was classified as left, right, or bilateral. To evaluate significance, we used the Fisher’s Exact Test (2 × 2 matrix, two-tailed).

#### Whole-mount *in situ* hybridization (WISH) of zebrafish embryos

RNA *in situ* hybridization was carried out according to standard protocol (Thisse and Thisse, 2008). Briefly, zebrafish embryos were fixed overnight in 4% paraformaldehyde at 4°C. Digoxigenin-UTP-labeled antisense probes for *spaw* and *lefty2* genes were used. Alkaline phosphatase-coupled anti-digoxigenin antibodies (Roche, #11093274910) were used to detect hybridized probes. NBT/BCIP solution (Roche, #11681460001) was used to visualize the signal under a stereomicroscope.

#### Immunofluorescence microscopy of zebrafish embryos

Zebrafish embryos were fixed in Dent’s fix (80% methanol, 20% DMSO), for at least 3 hr at room temperature or with fish fixative overnight at 4°C. Fixed embryos were stored in methanol at −20°C. Embryos were washed in a decreasing methanol:PBS gradient, followed by a PBS wash and blocking in PBDT (1% (w/v) BSA, 1% DMSO, 0.5% Triton X-100, PBS base) for 1 hr. Primary antibodies were added to PBDT and incubated with the embryos at 4°C overnight. Embryos were then washed in PBDT before incubating with fluorophore-conjugated secondary antibodies and DAPI (4′,6-diamidino-2-phenylindole; Invitrogen #D1306) for 3 hr at room temperature. The embryos were then stored in 70% glycerol, mounted, and imaged using an Olympus Fluoview Upright Confocal Microscope. Image acquisition and analysis was carried out using Olympus Fluoview FV10-ASW software. The primary antibodies used were: mouse anti-myosin heavy chain A4.1025 (Developmental Studies Hybridoma Bank, 1:20), rabbit anti-acetylated tubulin (Cell Signaling Technology #5335) and mouse anti-γ-tubulin GTU-88 (Sigma #T6557) (both 1:500). DAPI was used to label cell nuclei.

#### High-speed video microscopy of zebrafish cilia

To record cilia motility, zebrafish embryos were embedded in 2% agarose (with 0.0175% Tricaine for 24 hpf embryos) on 50 mm glass-bottom dishes. Ciliary motility was viewed with a 63X water-dipping objective on an upright Zeiss Axioplan2 microscope equipped with a Hamamatsu ORCA-Flash4.0 V2 C11440-22CU camera. Processing of videos was performed with ImageJ 1.44d (Schneider et al., 2012).

#### Transmission electron microscopy (TEM) of KV cilia

Zebrafish embryos at 10 somites stage (~14 hpf) were fixed with a standard TEM fixative solution of 2.5% glutaraldehyde and 4% paraformaldehyde in 0.1 M HEPES at 4°C overnight. Samples were washed with HEPES buffer and post fixed in 1% osmium tetroxide in 0.1 M HEPES for 2 hr. After post fixation, samples were rinsed with 0.1 M HEPES three times and treated with 1% tannic acid in 0.1 M HEPES buffer for 1 hr. Consecutively, samples were washed thoroughly with water three times. Samples were treated with series of ethanol solutions (50%, 75% ethanol) for ten minutes. Embryos were processed for enblock staining with 1% uranyl acetate in 75% ethanol for 1 hr on ice. After enblock staining, samples were processed for dehydration in a series of ethanol solutions (85%, 90%, 95%) on ice. The final dehydration procedure was done at room temperature using 100% ethanol twice for 15 minutes. Dehydration was continued with propylene oxide for 15 minutes two times. Subsequently, infiltration was done using propylene oxide and epon resin mixture. Later 100% epon was used for overnight infiltration. Embryos were changed into 100% fresh epon resin two times before embedding. Samples were embedded in 100% resin and polymerized at 64°C. Ultrathin sections, with thickness of 60 nm, were collected, stained with lead citrate solution, and imaged with a JEOL Flash-1400 microscope.

#### Mouse genotyping

All mice were genotyped at 3 weeks of age by collection of ~1 mm diameter ear clips. DNA was extracted by adding 20 μg Proteinase K, 50 mM Tris pH 8.0, 0.5% Tween, 1 mM EDTA and H_2_O to a total volume of 35 ml, followed by incubation at 55°C for 1 hour (for proteinase activity) and 95°C for 5 mins (to inactivate the enzyme). 50 ng of extracted DNA was used in downstream genotyping assays. Allele counts were determined via quantitative reverse transcription PCR (RT-qPCR). Embryos were genotyped by collection of either the yolk sac during dissection, or a tail segment after analysis and alleles were determined via PCR and amplicon visualization on agarose gels.

#### Quantitative reverse transcription PCR (RT-qPCR)

Adult mouse tissue was collected, and total RNA extracted (QIAGEN RNeasy mini kit, #74104). cDNA was synthesized using an Applied Biosystems High-Capacity cDNA Reverse Transcription Kit (Thermo Fisher Scientific, #4368814). Primers were designed to be specific to the gene of interest and their amplification efficiency was checked using testis cDNA. Only primers that produced a single amplicon were used for analysis. All RT-qPCR experiments used Agilent Technologies qPCR Brilliant IISYBR Master Mix (Cat #600828) and experiments were performed on an Applied Biosystems 7500 Fast Real-Time PCR machine. Gene expression was assessed using 50 ng of cDNA in technical triplicates and 5 biological replicates, with Gapdh used at the reference gene for normalization.

#### Antibody Generation

Recombinant 6xHis-tagged mouse PIERCE1 protein was expressed in BL21(DE3)pLysS competent cells (Novagen) and purified by Ni-NTA affinity chromatography. Purified protein was injected into New Zealand White rabbits (by Covalab, France); antibodies were purified from rabbit sera by affinity purification using PIERCE1 recombinant protein bound to SulfoLink resin (Thermo Fisher Scientific, Cat #20401).

#### Western Blot Analysis

Whole cell lysates from mouse testes were extracted with SDS lysis buffer, containing protease inhibitor cocktail, on ice by homogenization. The homogenates were incubated on ice for 30 min, and cell debris was pelleted by centrifugation at 13,000 rpm, at 4°C for 10 min. Supernatant samples were resolved on 12% SDS-PAGE gels and transferred onto polyvinylidene fluoride (PVDF) membrane (Pall Corporation). Following transfer, membranes were blocked with 5% non-fat milk dissolved in 1X Tris Buffered Saline-Tween (TBS-TWEEN) for 1 hr at room temperature. Primary antibody (rabbit anti-Pierce1, polyclonal, 1:200), diluted in the same blocking solution, was added to the membrane, and incubated overnight at 4°C. The membrane was washed 3 times with TBS-TWEEN at 10 min intervals. Secondary antibody (polyclonal goat anti-rabbit conjugated with horseradish peroxidase (HRP), Agilent Cat #P0448), diluted (1:2000) in blocking solution, was added to the membrane and incubated for 1 hr at room temperature, followed by three TBS-TWEEN washes at 10 min intervals. Enhanced chemiluminescent (ECL) substrate (Geneflow) was used to detect the signal from the HRP-conjugated secondary antibody.

#### Mouse embryo phenotyping

Embryos were dissected in phosphate buffered saline (PBS) under a light microscope. The head was removed, and the thoracic cavity opened. For phenotyping, embryos were scored according to lung lobation, heart apex position, heart outflow tract patterning and stomach position. Any other gross abnormalities were also noted. All imaging was performed in PBS using a Teledyne Lumenera Infinity3-6URC camera on a Leica MZ12.5 microscope. Tail clips were taken after imaging for genotyping purposes.

#### LacZ embryo staining

Embryos were harvested in PBS at the desired developmental time point and fixed in 1% formaldehyde, 0.2% glutaraldehyde, 2 mM MgCl_2_, 5 mM EGTA and 0.02% NP-40 in PBS overnight. Samples were washed 3× with 0.02% NP-40 in PBS and stained for 18 hours at 37°C with 0.5 mg/ml X-gal, 10 mM K_3_Fe(CN)_6_, 10 mM K_4_Fe(CN)_6_, 2 mM MgCl_2_, 0.01% sodium deoxycholate and 0.02% NP-40 in PBS. Stained embryos were imaged in PBS using a Leica DFC420 camera on a Leica MZ16F microscope. Whole embryos were taken after imaging for genotyping purposes.

#### Nodal cilia analysis

Embryos from *Pierce1*^+/−^ × *Pierce1*^+/−^ or *Pierce2*^+/−^ × *Pierce2*^+/−^ crosses were harvested at E8.0 and dissected in pre-warmed DMEM (GIBCO, #10569010) supplemented with 10% FBS (GIBCO, #10500064). To visualize nodal cilia rotation, embryos were mounted on slides in the medium with the node facing up. Differential interference contrast (DIC) video capture was performed at 100 frames per second at 100X magnification using a Leica DM2500 compound microscope equipped with a monochrome high-speed Hamamatsu C9300 camera. Nodal cilia rotation was quantified by counting the number of frames per 5 complete rotations for a minimum of 5 cilia per embryo. Cilia movement type was also determined from the same videos. Embryos were taken for genotyping after imaging.

#### Particle image velocimetry (PIV) of nodal cilia

Embryos from *Pierce1*^+/−^ × *Pierce1*^+/−^ intercrosses were harvested at E8.0 in pre-warmed DMEM with 10% FBS. 0.2 μm diameter FluoSpheres (Thermo Fisher Scientific, #F8848) were diluted 1:10 with the same DMEM with 10% FBS medium and then placed over embryos mounted on pre-warmed (37°C) glass microscope slides. Videos were captured using a Zeiss EC Plan-Neofluar 40×/0.75 lens and an AxioCam HRm camera with a VivaTome attachment, on a Zeiss Observer.Z1 microscope. Data points were binarized and dilated using ImageJ (Schneider et al., 2012), before particle image velocimetry analysis using PIVLab plugin in MATLAB (Thielicke and Stamhuis, 2014) to track the movement of each fluorescent bead frame-to-frame. Embryos at 2-3 somite stages were used for this analysis.

#### Whole mount *in situ* hybridization (WISH) of mouse nodal genes

Anti-sense WISH probes for *Pierce1* and *Pierce2* were generated against DNA sequences corresponding to *Pierce1* 16-768 nt (NM_027040.1) and *Pierce2* 98-550 nt (NM_001198789.1). PCR-generated sequences were ligated into the pBluescript II KS(−) vector linearized with EcoRV. The identity of the cloned sequence was confirmed by DNA sequencing (Source BioScience) primed using the T3 and T7 promoter sequences. Digoxygenin-labeled anti-sense riboprobes for *Cerl2* (Marques et al., 2004), *Pitx2* (Ryan et al., 1998), *Pierce1*, and *Pierce2* were transcribed from either the T3 or T7 promoter. WISH experiments were performed as described (Field et al., 2011) using anti-Digoxigenin antibody (Roche, #11093274910) and NBT/BCIP staining (Roche, #11681460001). Stained embryos were imaged in PBS using a Leica DFC420 camera on a Leica MZ16F microscope. Whole embryos were taken after imaging for genotyping.

#### High-speed video microscopy of tracheal motile cilia

Tracheas from 10–12-week-old mice were harvested, excess tissue removed, then cut into rings approximately the width of one cartilage ridge. Samples were incubated at 37°C, 5% CO_2_ overnight in MEM (GIBCO, #11544456) medium with 1% penicillin-streptomycin (GIBCO, #11528876) and 0.2% nystatin (GIBCO, #11548886). Rings were allowed to settle for 20 mins on the stage of an inverted Olympus IX71 microscope in an environmental chamber at 37°C, prior to video capture. High-speed video recordings were taken at 500 frames per second, 60X magnification objective (Olympus LUCPlan FLN) using a Photron MC 2.1 FastCam camera. Average ciliary beat frequency (CBF) was measured by fast Fourier transform analysis using a CBF panel FFT V2.700 ImageJ plugin. Ciliary beat pattern (CBP, stiffness), amplitude and synchronization were independently assessed by 3 expert reviewers who were blind to sample genotypes, viewing videos at 30 frames per second. Videos containing debris or non-ciliated cells were excluded from the analysis.

#### TEM of mouse tracheal cilia

Tracheas were harvested as detailed above. Rings were fixed in 3% glutaraldehyde, 0.1 M cacodylate buffer (overnight), post-fixed in 2% osmium tetroxide in 0.1 M cacodylate buffer (2 hours), dehydrated in ethanol and embedded in Spurr’s resin (Spurr, 1969) following an established protocol. Ultra-thin sections (100 nm) were cut (Leica EM UC7 ultramicrotome) and stained with lead citrate, then imaged using a Hitachi HT7700 transmission electron microscope. Ultrastructural defects were scored by examination of 300+ ciliary cross-sections per sample by an expert electron microscopist. Acilium was tagged as defective for axonemal dyneins if fewer than 7 ODAs or 5 IDAs were observed.

#### Figures

Figure panels depicting cryo-EM maps or atomic models were generated using Chimera (Pettersen et al., 2004) or ChimeraX (Pettersen et al., 2021). Maps colored by local resolution were generated using RELION 3.1 (Zivanov et al., 2018). Structural biology software were installed and configured by SBGrid (Morin et al., 2013).

### QUANTIFICATION AND STATISTICAL ANALYSIS

Resolution estimations of cryo-EM density maps are based on the 0.143 FSC criterion (Rosenthal and Henderson, 2003). All statistical validation performed on the deposited model (PDB: 7RRO) was done using the PHENIX package (Table S2). Statistical analysis in Figure 4E was performed with Microsoft Excel (Microsoft Corporation). Statistical analyses in Figures 6 and 7 and S6 were performed with GraphPad Prism v9 (GraphPad Software). Further details can be found in the corresponding figure legends.

## Supplementary Material

1

2

4

6

3

8

9

10

7

12

5

13

11

14

## Figures and Tables

**Table:** KEY RESOURCES TABLE

REAGENT or RESOURCE	SOURCE	IDENTIFIER
Antibodies		
Mouse anti-myosin heavy chain A4.1025, unconjugated	Developmental Studies Hybridoma Bank	DSHB Cat #A4.1025; RRID:AB_528356
Sheep anti-Digoxigenin Fab fragments antibody, alkaline phosphatase conjugated	Roche	Cat #11093274910; RRID:AB_514497
Rabbit anti-acetylated tubulin D20G3, unconjugated	Cell Signaling Technology	Cat #5335; RRID:AB_10544694
Mouse anti-γ-tubulin GTU-88, unconjugated	Sigma	Cat #T6557; RRID:AB_477584
Rabbit anti-Pierce1, polyclonal, unconjugated	This study	N/A
Goat anti-rabbit, polyclonal, HRP conjugated	Agilent	Cat #P0448; RRID:AB_2617138
Biological samples		
Bovine trachea	Adam’s Farm (Athol, MA)	N/A
Chemicals, peptides, and recombinant proteins		
1,4-Dithiothreitol (DTT)	Roche	Cat #DTT-RO
3-[(3-Cholamidopropyl)dimethylammonio]-1-propanesulfonate (CHAPS)	Sigma	Cat #C3023-5G
4′,6-diamidino-2-phenylindole (DAPI)	Invitrogen	Cat #D1306
Adenosine 5′-triphosphate (ATP)	Sigma	Cat #A9187
cOmplete Protease Inhibitor Cocktail	Roche	Cat #CO-RO
Dulbecco’s Modified Eagle Medium (DMEM), high glucose with GlutaMAX	GIBCO	Cat #10569010
Ethylenediaminetetraacetic acid (EDTA)	Oakwood Chemical	Cat #238173
Fetal Bovine Serum (FBS)	GIBCO	Cat #10500064
FluoSpheres Sulfate Microsphere, 0.2 μm	Invitrogen	Cat #F8848
Haematoxylin solution Gill No. 3	Sigma-Aldrich	Cat #GHS332
MEM, HEPES, no glutamine	GIBCO	Cat #11544456
Nystatin, anti-fungal agent	GIBCO	Cat #11548886
Phusion High-Fidelity DNA polymerase	NEB	Cat #M0530S
Penicillin/streptomycin	GIBCO	Cat #11528876
ProteaseArrest protease inhibitor cocktail	G Bioscience	Cat #786-108
NBT/BCIP solution	Roche	Cat #11681460001
NP-40 detergent	Thermo Fisher Scientific	Cat #85124
Recombinant mouse PIERCE1	This study	N/A
*S. pyogenes* Cas9 protein	Toolgen	Cat #TGEN_CP1
Sarkosyl	IBI Scientific	Cat #IB07080
Sodium citrate	BDH Chemicals Ltd	Cat #10242
Sodium Hydroxide	Fisher Scientific	Cat #S/4920/60
SulfoLink coupling resin	Thermo Fisher Scientific	Cat #20401
Trizol	Ambion Life Technologies	Cat #15596018
Trypsin, sequencing-grade	Promega	Cat #V5111
Uranyl formate	Electron Microscopy Sciences	Cat # 22451
Critical commercial assays		
Agilent Technologies qPCR Brilliant II SYBR Master Mix	Agilent Technologies	Cat #600828
DIG RNA labeling mix	Roche	Cat #11277073910
High-Capacity cDNA Reverse Transcription Kit	Applied Biosystems	Cat #4368814
MEGAshortscript T7 Transcription Kit	Ambion	Cat #AM1354
Pierce Silver Stain Kit	Thermo Fisher Scientific	Cat #24612
QIAGEN RNeasy mini kit	QIAGEN	Cat #74104
RT-PCR Kit	QIAGEN	Cat #210212
Taq PCR Master Mix Kit	QIAGEN	Cat #201445
TaqMan GTXpress Master Mix	Applied Biosystems	Cat #4401892
T3 transcription kit	Thermo Scientific	Cat #EP0101
T7 transcription kit	Thermo Scientific	Cat #EP0112
Deposited data		
Composite cryo-EM density map of the 48-nm repeat of the doublet microtubule from *Bos taurus*	This paper	EMDB: EMD-24664
Cryo-EM density map of the ODA core from *Bos taurus*	This paper	EMDB: EMD-24663
Atomic model of the 48-nm repeat of the doublet microtubule from *Bos taurus*	This paper	PDB: 7RRO
Atomic Model of the *Chlamydomonas* doublet microtubule	Ma et al., 2019	PDB: 6U42
Atomic Model of the *Chlamydomonas* ODA-DC	Walton et al., 2021	PDB: 7KZO
Atomic Model of the *Chlamydomonas* ODA	Walton et al., 2021	PDB: 7KZM
Subtomogram average of the bovine doublet microtubule	Greenan et al., 2020	EMDB: EMD-20674
Subtomogram average of the human doublet microtubule	Lin et al., 2014b	EMDB: EMD-5950
Subtomogram average of the zebrafish doublet microtubule	Yamaguchi et al., 2018	EMDB: EMD-6954
Subtomogram average of the *Chlamydomonas* doublet microtubule	Owa et al., 2019	EMDB: EMD-9768
Subtomogram average of the *Tetrahymena* doublet microtubule	Imhof et al., 2019	EMDB: EMD-20012
Subtomogram average of the Chinese hamster ovary centriole	Greenan et al., 2018	EMDB: EMD-7775
Subtomogram average of the bovine basal body	Greenan et al., 2020	EMDB: EMD-20676
Experimental models: Organisms/strains		
Bacteria: *Escherichia coli* DH5α Competent Cells	Thermo Fisher Scientific	Cat #18265017
Bacteria: *Escherichia coli* DH5α Competent Cells	New England Biolabs	Cat #C2987H
Zebrafish: AB wild-type strain	Zebrafish International Resource Center (ZIRC)	RRID:ZIRC_ZL1
Zebrafish: *pierce1* KO c.178_179ins(29 bp)	This paper	N/A
Zebrafish: *pierce2* KO c.166_167ins(34bp)	This paper	N/A
Zebrafish: *pierce1*^*+2*^ *; pierce2*^*+34*^ double knockout	This paper	N/A
Mouse: *Pierce1*^−/−^ *(1700007K13Rik^tm2b(EUCOMM)Wtsi^*)	MGI	RRID:MGI:5756622
Mouse: *Pierce2*^−/−^ (*Ccpg1os*^em1(IMPC)H^)	This paper	N/A
Mouse: *Pierce1*^−/−^ *; Pierce2*^−/−^ double knockout	This paper	N/A
Oligonucleotides		
A full list is provided in Table S4		
Recombinant DNA		
Plasmid: pCR II-TOPO	Invitrogen	N/A
Plasmid: pBluescript II KS(-)	Stratagene	Cat #212208
Software and algorithms		
BLAST	Altschul et al., 1990	https://blast.ncbi.nlm.nih.gov/Blast.cgi
CCP4 suite	Winn et al., 2011	https://www.ccp4.ac.uk/
Chimera v1.13.1 or v1.14	Pettersen et al., 2004	https://www.cgl.ucsf.edu/chimera/
ChimeraX v1.1	Pettersen et al., 2021	https://www.rbvi.ucsf.edu/chimerax/
CHOPCHOP v3	Montague et al., 2014	https://chopchop.cbu.uib.no/
Clustal Omega v1.2.2	Sievers et al., 2011	http://www.clustal.org/omega/
CMView v1.1.1	Vehlow et al., 2011	http://www.bioinformatics.org/cmview/
Coot v0.9-pre or v0.9.4.1	Emsley and Cowtan, 2004	https://www2.mrc-lmb.cam.ac.uk/personal/pemsley/coot/
CTFFIND4	Rohou and Grigorieff, 2015	https://grigoriefflab.umassmed.edu/ctffind4
DeepEMhancer	Sánchez-García et al., 2020	https://github.com/rsanchezgarc/deepEMhancer
ESPript 3.0	Robert and Gouet, 2014	https://espript.ibcp.fr/
EV-couplings V2 server	Hopf et al., 2019	https://v2.evcouplings.org/
ImageJ 1.44d	Schneider et al., 2012	https://imagej.github.io/
MOLREP v11.6	Vagin and Teplyakov, 2010	https://www.ccp4.ac.uk/html/molrep.html
MotionCor2	Zheng et al., 2017	https://msg.ucsf.edu/software
phenix.auto_sharpen v1.18.2-3874	Afonine et al., 2018	https://phenix-online.org/
phenix.molprobity v1.18.2-3874	Chen et al., 2010	https://phenix-online.org/
phenix.real_space_refine v1.18.2-3874	Afonine et al., 2018	https://phenix-online.org/
PIVLab plugin for MATLAB	Thielicke and Stamhuis, 2014	https://www.mathworks.com/matlabcentral/fileexchange/27659-pivlab-particle-image-velocimetry-piv-tool-with-gui
Prism v9	GraphPad	https://www.graphpad.com
PSIPRED 4.0	Jones, 1999	http://bioinf.cs.ucl.ac.uk/psipred/
RELION-3.1	Zivanov et al., 2018	https://www3.mrc-lmb.cam.ac.uk/relion/
SBGrid	Morin et al., 2013	https://sbgrid.org/
SerialEM 3.7	Schorb et al., 2019	https://bio3d.colorado.edu/SerialEM/
SEQUEST	Thermo Fisher Scientific	https://www.thermofisher.com/us/en/home.html
SWISS-MODEL	Waterhouse et al., 2018	https://swissmodel.expasy.org/
TrRosetta	Yang et al., 2020	https://robetta.bakerlab.org
Python 3.8.8	Python Software Foundation	https://www.python.org/downloads/release/python-388/
Other		
C-flat holy carbon grids (R1.2/1.3, 400 mesh copper)	Electron Microscopy Sciences	Cat #CF413-50
Quantifoil holy carbon grids (R1.2/1.3, 400 mesh gold)	Quantifoil Micro Tools	Cat #Q4100AR1.3
Quantifoil holy carbon grids (R2/2, 400 mesh copper)	Quantifoil Micro Tools	Cat #Q4100CR2
Curated list of tektin sequences	Bastin and Schneider, 2019	N/A
Rat and Mouse Diet No. 3	Special Diet Services, UK	Cat #RM3

## Highlights

- Structure of the 48-nm repeat of a mammalian doublet microtubule from respiratory cilia
- Tektin filaments bind within the lumens of doublet microtubules
- A pentameric docking complex attaches axonemal dyneins to doublet microtubules
- Loss of connectivity across microtubule walls causes ciliopathy-like phenotypes
