# Supplementary material for: De novo identification of mammalian ciliary motility proteins using cryo-EM
Source: Cell. Author manuscript; Available in PMC 2021 Nov 17. (PMC8595878; doi:10.1016/j.cell.2021.10.007)
Supplement: 2 [file NIHMS1749474-supplement-2.pdf]

SUPPLEMENTAL TABLES

Table S2. Statistics for data collection, data processing, model refinement and validation, Related to Figure 1.

	**Composite map of 48-nm repeat (EMD-24664, PDB ID 7RRO)**	**ODA core (EMD-24663)**
**Data collection**		
Microscope	Titan Krios	Titan Krios
Detector	K3	K3
Voltage (keV)	300	300
Nominal magnification	81,000×	81,000×
Electron exposure ($e^-/Å^2$)	60	60
Defocus range set during data acquisition (μm)	-1.0 to -2.5	-1.0 to -2.5
Pixel size (Å)	1.09	1.09
**Data Processing**		
Particles	80,503	8,755
Map resolution (Å)	3.4	8.0
**Model composition**		
Chains	431	
Atoms	1,276,295	
Residues	161,535	
Ligands	149 GTP / 153 GDP / 149 Mg	
**Refinement**		
Resolution limit set in refinement (Å)	3.6	
Correlation coefficient (CCmask)	0.71	
C_{ref} (masked) (Å)	3.7	
Root-mean-square deviation (bond lengths) (Å)	0.008	
Root-mean-square deviation (bond angles) (Å)	1.196	
**Validation**		
MolProbity Score	1.85	
Clashscore	7.23	
Rotamer outliers (%)	0.24	
Ramachandran (favored) (%)	92.82	
Ramachandran (outliers) (%)	0.0	

Table S3. Proteins associated with ciliopathies, Related to Figures 1, 6 and 7.

Axonemal protein	Human disease association	Mouse knockout phenotype	Zebrafish phenotype
**ODA-DC**			
ARMC4 (ODAD2)	Laterality abnormalities, recurrent airway infections, bronchiectasis, neonatal respiratory distress, ODA loss, reduced ciliary beat (Hjeij et al., 2013; Onoufriadis et al., 2014).	Missense mutants (M993K) have laterality abnormalities, immotile tracheal cilia, decreased fluid flow by ependymal cilia, and ODA loss (Hjeij et al., 2013).	*armc4* morphants have laterality abnormalities (Hjeij et al., 2013).
Calaxin (EFCAB1, ODAD5)		Laterality defects, abnormal sperm motility, decreased mucociliary flow rates (Sasaki et al., 2019).	*calaxin* knockout mutants have laterality abnormalities (Sasaki et al., 2019).
CCDC114 (ODAD1)	Laterality abnormalities, recurrent airway infections, neonatal respiratory distress, ODA loss, immotile cilia (Chen et al., 2020; Knowles et al., 2013; Onoufriadis et al., 2013; Wu and Singaraja, 2013).		
CCDC151 (ODAD3)	Laterality abnormalities, (Hjeij et al., 2014; Zhang et al., 2019).	Perinatal lethality and congenital hydrocephalus, defective sperm motility (Chiani et al., 2019).<br><br>*Ccdc151*^{Snbl} mutants have laterality abnormalities, immotile tracheal cilia and ODA loss (Hjeij et al., 2014).	*ccdc151* morphants have laterality abnormalities, kidney cysts and immotile cilia (Jerber et al., 2014).
TTC25 (ODAD4)	Laterality abnormalities, recurrent airway infections, neonatal respiratory distress, ODA loss, immotile airway cilia (Wallmeier et al., 2016).	Laterality abnormalities (Wallmeier et al., 2016).	*ttc25* morphants have body curvature, hydrocephalus, and pronephric cyst formation (Xu et al., 2015).<br><br>*Ttc25*^{ts272a} mutants have body curvature and kidney cysts (Hjeij et al., 2014).
**Luminal proteins (MIPs)**			
CFAP45	Laterality abnormalities and asthenospermia. Mild respiratory symptoms (Dougherty et al., 2020).	Laterality abnormalities and asthenospermia (Dougherty et al., 2020).	
CFAP52 (WDR16)	Laterality abnormalities (Ta-Shma et al., 2015).		*cfap52* morphants have severe hydrocephalus (Hirschner et al., 2007).
CFAP53 (MBD1, CCDC11)	Laterality abnormalities (Narasimhan et al., 2015; Noël et al., 2016; Perles et al., 2012).	Laterality abnormalities, hydrocephalus, abnormal airway cilia, infertility, ODA loss in nodal cilia (Ide et al., 2020).	*cfap53* morphants have laterality abnormalities and body curvature (Narasimhan et al., 2015).<br><br>*cfap53* mutants have disrupted laterality without additional morphological defects (Noël et al., 2016; Silva et al., 2016).
CFAP161 (C15orf26)			*Cfap161* morphants have laterality defects, body curvature and ODA loss (Austin-Tse et al., 2013).
CFAP276 (C1orf194)	Associated with Charcot-Marie-Tooth disease (Sun et al., 2019).	Motor and sensory defects (Huang et al., 2020).	
EFHC1	Association with juvenile myoclonic epilepsy is debated (Gonsales et al., 2020).	Ventricle enlargement, reduced beat frequency of ependymal cilia, spontaneous myoclonus (Suzuki et al., 2009).	
EFHC2			*efhc2* morphants have defects in pronephros development (Barrodia et al., 2018).
ENKUR		Subfertility and abnormal sperm motility (Jungnickel et al., 2018).	
FAM166B	Associated with Meckel-Gruber syndrome (Shamseldin et al., 2020).		
MNS1	Laterality abnormalities, infertility, oligoasthenoteratozoospermia, reduced ODA levels (Leslie et al., 2020; Li et al., 2020; Ta-Shma et al., 2018).	Laterality abnormalities, hydrocephalus, infertility, reduced ODA levels (Zhou et al., 2012).	
NME7	Laterality abnormalities (Reish et al., 2016).	Laterality abnormalities (Vogel et al., 2010).	

PACRG		Male sterility (Lorenzetti et al., 2004). Reduction in ependymal cilia beat frequency and cilia-mediated flow (Wilson et al., 2010).	
Pierce1 (C9orf116)		Laterality abnormalities, partial embryonic lethality associated with heterotaxia (Sung et al., 2016). Reduced airway and nodal cilia beat frequency (this study).	*Pierce1 and C18h15orf65* double mutants have heart jogging defects, body curvature, and immotile KV cilia (this study).
Pierce 2 (C15orf65)		Reduced nodal cilia beat frequency (this study).	*Pierce1* and *Pierce2* double mutants have heart jogging defects, body curvature and immotile KV cilia (this study).
Tektin 1	Potentially associated with primary ciliary dyskinesia (Ryan et al., 2018).		*tekt1* morphants have mild body curvature, laterality defects, and cyst formation in the pronephros (Ryan et al., 2018).
Tektin 2		Male infertility, reduced sperm motility, reduced mucociliary flow (Tanaka et al., 2004).	
Tektin 3		Reduced sperm motility, sperm flagella abnormalities (Roy et al., 2009).	
Tektin 4		Subfertility, sperm with reduced motility (Roy et al., 2007).	

Table S4. Oligonucleotides used in this paper, Related to STAR Methods.

Oligonucleotides	Sequence (5' to 3')	Source	Identifier
Zebrafish pierce1 sgRNA forward primer:	GAAATTAATACGACTCACTATATGGTTTGTTGTTCGGTACAGGTTTTAGAGCTAGAAATA	Integrated DNA Technologies, Inc. (IDT)	N/A
Zebrafish pierce2 sgRNA forward primer:	GAAATTAATACGACTCACTATATCCAGCGTCGCGGGTCGAGCGCCGGTTTTAGAGCTAGAAATA	IDT	N/A
Zebrafish gRNA reverse:	AAAAGCACCGACTCGGTGCCACTTTTTCAAGTTGATAACGGACTAGCCTTATTTTAACTTGCTATTTCTAGCTCTAAAAC	IDT	N/A
Zebrafish pierce1 genotyping forward primer:	TACTCACAGGCATTTCGTGAAC	IDT	N/A
Zebrafish pierce1 genotyping reverse primer:	TGAGTTTGAAATCAAAACAAGCA	IDT	N/A
Zebrafish pierce2 genotyping forward primer:	ATGTCGAACTCTGCGCATC	IDT	N/A
Zebrafish pierce2 genotyping reverse primer:	TCTGTTTAAAGGACAGTGA	IDT	N/A
Mouse Pierce1 gel-based genotyping (WT & Tm2b alleles):	ACAGGAGCTTGGCCTCTGTA	Merck Life Science	N/A
Mouse Pierce1 gel-based genotyping (WT allele):	AGAGCACTCTCTCCCAGCAG	Merck Life Science	N/A
Mouse Pierce1 gel-based genotyping (Tm2b allele):	GAACTTCGGAATAGGAACTTCG	Merck Life Science	N/A
Mouse Pierce1 Taqman genotyping (WT allele):	GGCTGCTTTTGCACTTGAAAC	LGC Biosearch Technologies	N/A
Mouse Pierce1 Taqman genotyping (WT allele):	CCAGCCATGCACGAAAAGTTC	LGC Biosearch Technologies	N/A
Mouse Pierce1 Taqman genotyping (Tm2b allele):	CTCGCCACTTCAACATCAAC	LGC Biosearch Technologies	N/A
Mouse Pierce1 Taqman genotyping (Tm2b allele):	TTATCAGCCGGAAAACCTACC	LGC Biosearch Technologies	N/A
Mouse Pierce2 gel-based genotyping (WT & DEL alleles):	GATAAGAAAACTTCCTTGAC	Merck Life Science	N/A
Mouse Pierce2 gel-based genotyping (WT & DEL alleles):	TGTTTTGAGAATATGCATTTG	Merck Life Science	N/A
Mouse Pierce2 Taqman genotyping (WT allele):	TTGGATCCCAAGACATTGC	LGC Biosearch Technologies	N/A
Mouse Pierce2 Taqman genotyping (WT allele):	TCTTGTACATAATCATCTGGGCTTT	LGC Biosearch Technologies	N/A
Mouse Pierce1 RT-qPCR (WT allele):	CATGGCTACGGGACCAAGGA	Merck Life Science	N/A
Mouse Pierce1 RT-qPCR (WT allele):	CCAAAAGCTGCGTGTTGTCT	Merck Life Science	N/A
Mouse Pierce1 RT-qPCR (Tm2b allele):	GAAAAACCTCAACAGTCCG	Merck Life Science	N/A
Mouse Pierce1 RT-qPCR (Tm2b allele):	CGGGACCTGGGACCCGTAG	Merck Life Science	N/A
Mouse Pierce2 RT-qPCR (WT & DEL alleles):	CAGACTGCGACTTGGATAAGAAA	Merck Life Science	N/A
Mouse Pierce2 RT-qPCR (WT allele):	CTTTGGACAGTGAGGTGGCT	Merck Life Science	N/A
Mouse Pierce2 RT-qPCR (DEL allele):	CTTATACCCCCACAGAGCCG	Merck Life Science	N/A
Mouse Pierce1 WISH probe plasmid generation:	CTGTCAGCATCTTTCCTGTGTG	Merck Life Science	N/A
Mouse Pierce1 WISH probe plasmid generation:	GCACAGACTTGATCTGGGCTA	Merck Life Science	N/A

Mouse Pierce2 WISH probe plasmid generation:	ACTCCGATCGTCTTGCTGTT	Merck Life Science	N/A
Pierce2 WISH probe plasmid generation:	TCGATGGTTCTGGTTCGGTC	Merck Life Science	N/A
Mouse WISH plasmid sequencing (T7):	TAATACGACTCACTATAGGG	Merck Life Science	N/A
Mouse WISH plasmid sequencing (T3):	ATTAACCCTCACTAAAG	Merck Life Science	N/A
Mouse Gapdh RT-qPCR:	CGGCCGCATCTTCTTGTG	Merck Life Science	N/A
Mouse Gapdh RT-qPCR:	CCGACCTTCACCATTTTGTCTA	Merck Life Science	N/A
