# Supplementary figures and images for: De novo identification of mammalian ciliary motility proteins using cryo-EM
Source: Cell. Author manuscript; Available in PMC 2021 Nov 17. (PMC8595878; doi:10.1016/j.cell.2021.10.007)

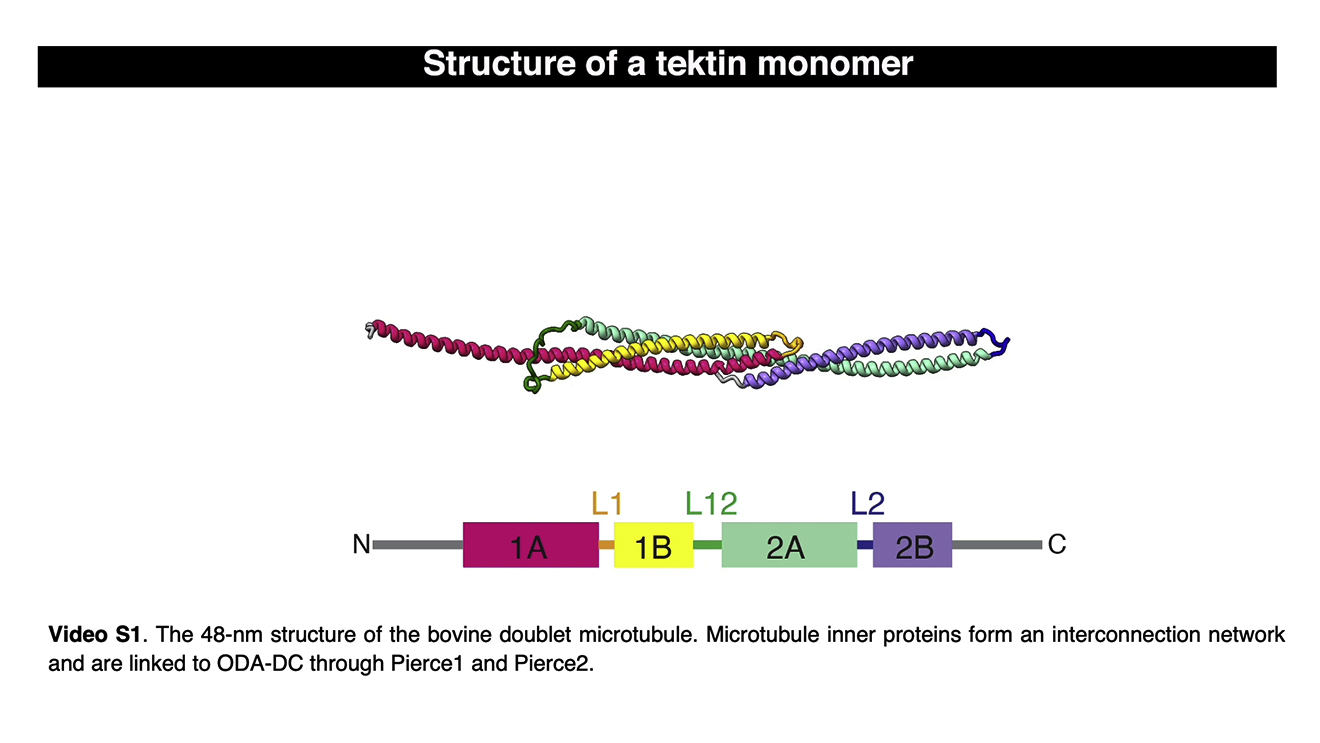

Supplement: 4 [file NIHMS1749474-supplement-4.jpg]

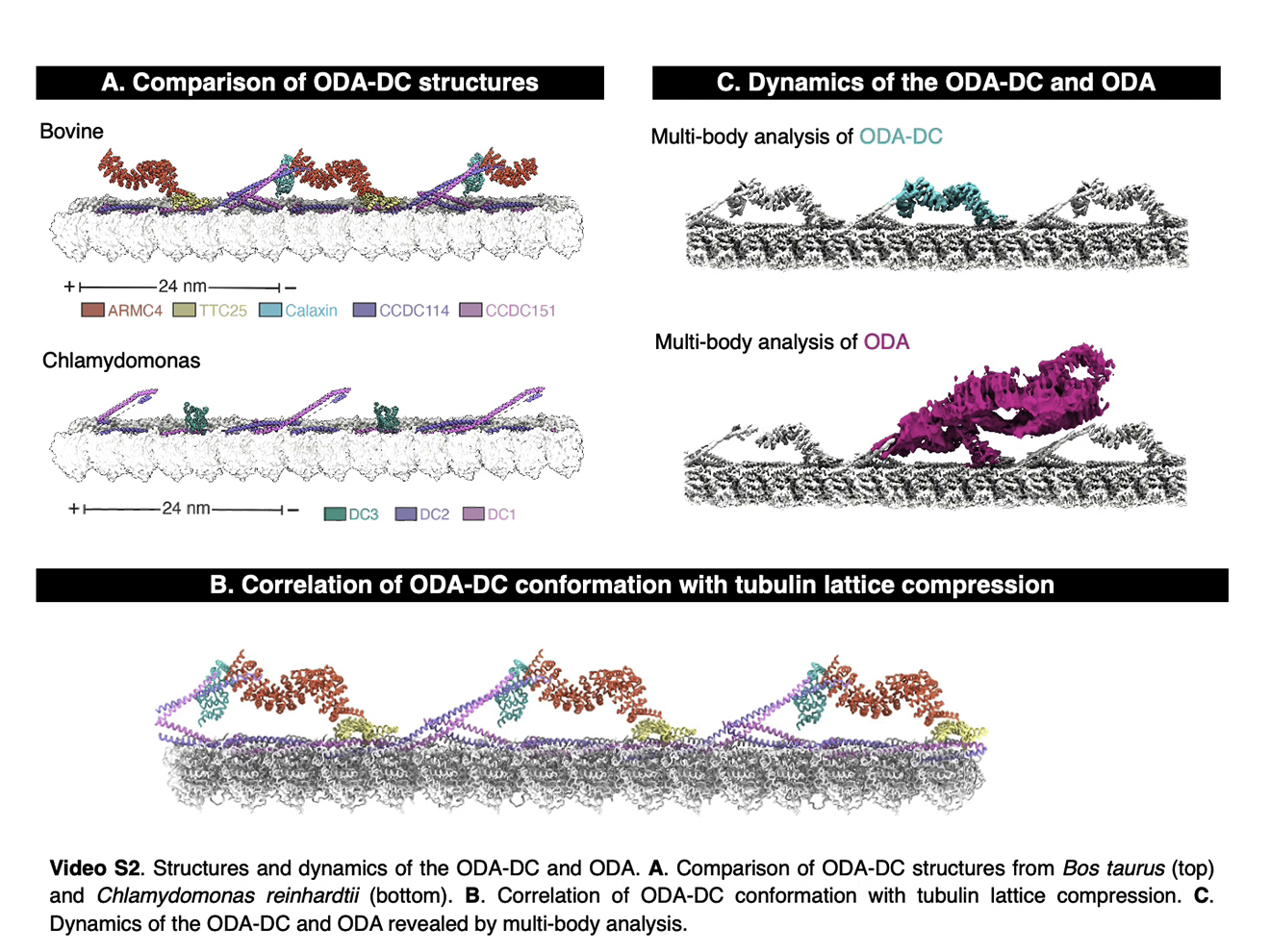

Supplement: 6 [file NIHMS1749474-supplement-6.jpg]

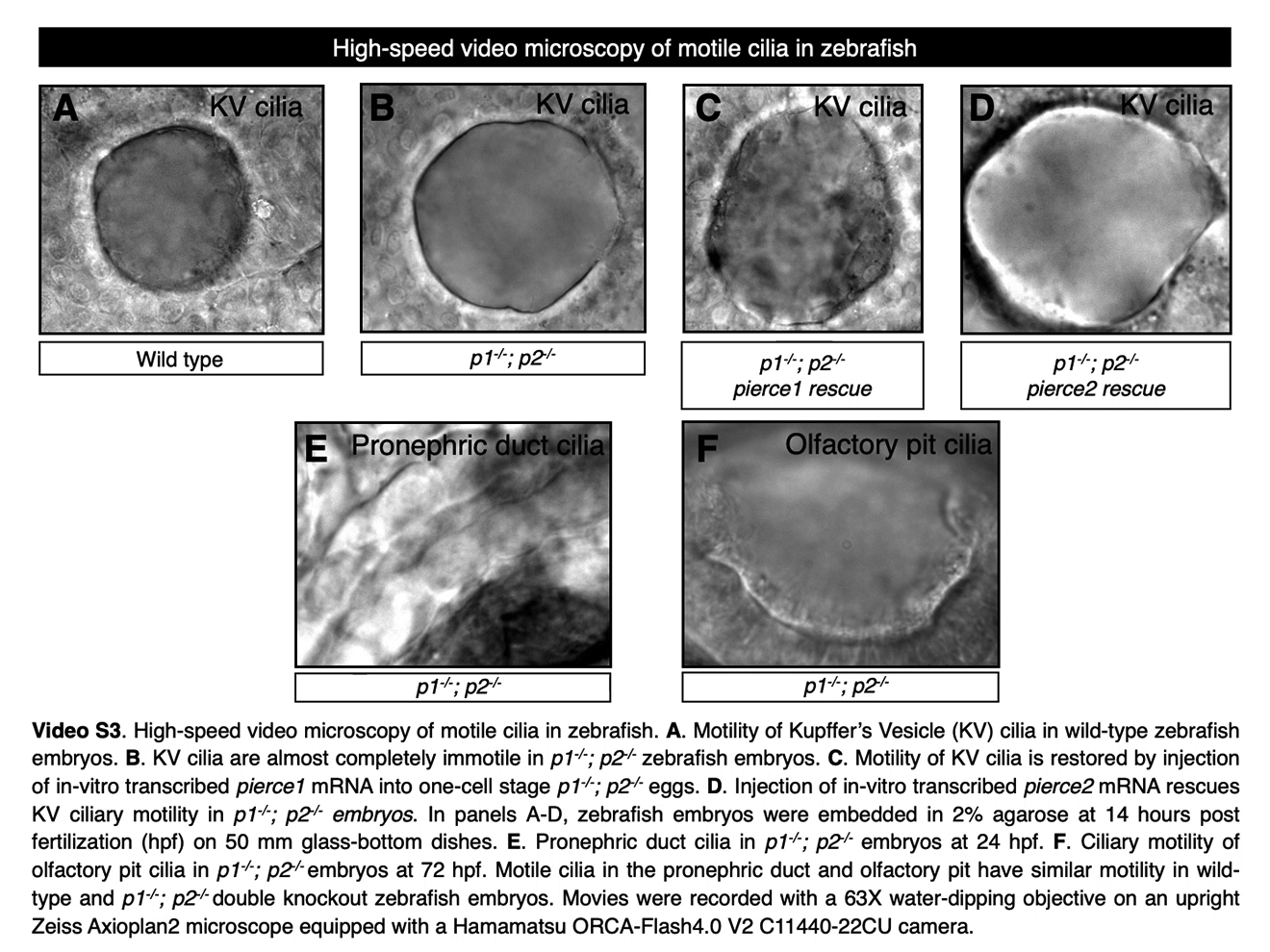

Supplement: 8 [file NIHMS1749474-supplement-8.jpg]

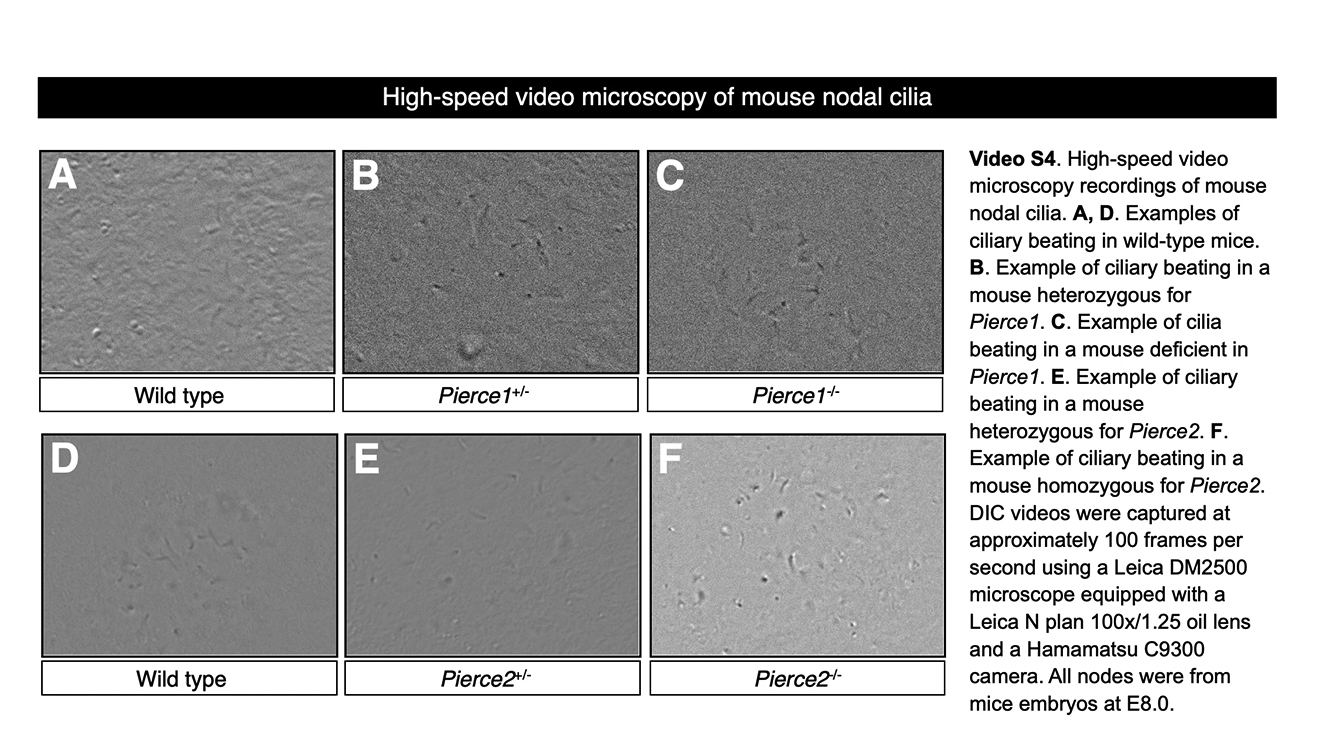

Supplement: 10 [file NIHMS1749474-supplement-10.jpg]

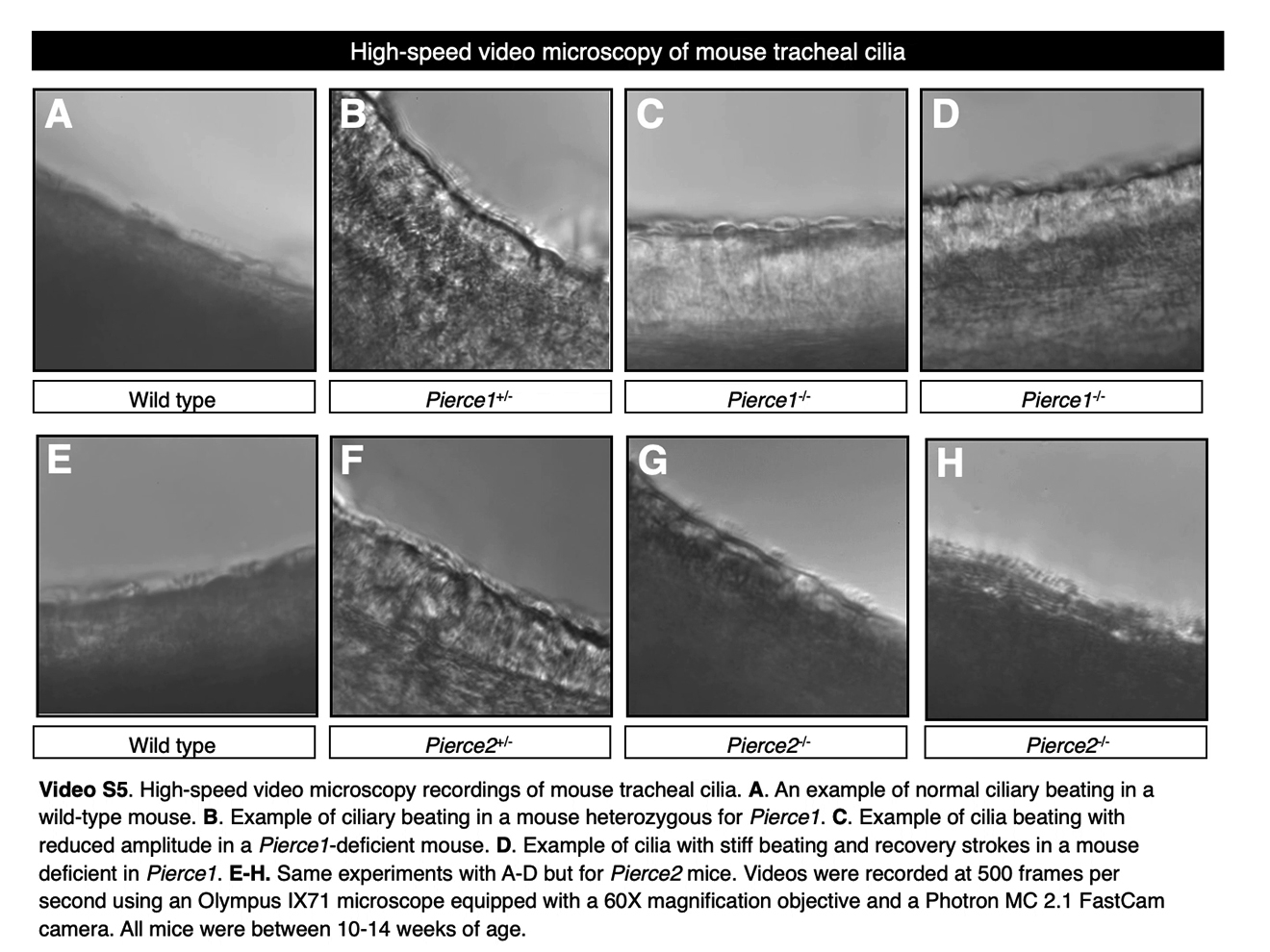

Supplement: 12 [file NIHMS1749474-supplement-12.jpg]
