# Supplementary material for: De novo identification of mammalian ciliary motility proteins using cryo-EM
Source: Cell. Author manuscript; Available in PMC 2021 Nov 17. (PMC8595878; doi:10.1016/j.cell.2021.10.007)
Supplement: 3 [file NIHMS1749474-supplement-3.pdf]

Methods S1

Cryo-EM data processing, Related to Figure 1.

Contents

Processing scheme 1: 8 to 48 nm p.2
Processing scheme 2: 48 to 96 nm p.3
Processing scheme 3: Determining a map of the ODA p.4
Processing scheme 4: Lattice classification p.5
Processing scheme 5: Tektin classification p.6
Processing scheme 6: Focused protofilament refinement/classification p.7
Processing scheme 7: Determining a map of the ODA-DC p.8
Processing scheme 8: Determining maps of Pierce1 and Pierce2 p.9
Examples of map quality for all identified proteins p.10
Proteins of the bovine DMT identified using cryo-EM p.11

Key for all figures

RX = re-box
SC = shift center
SS = signal subtraction
RD = remove duplicate particles
3C = 3D classification
3R = 3D refinement
MBR = multi-body refinement

Processing scheme 1: 8 to 48 nm

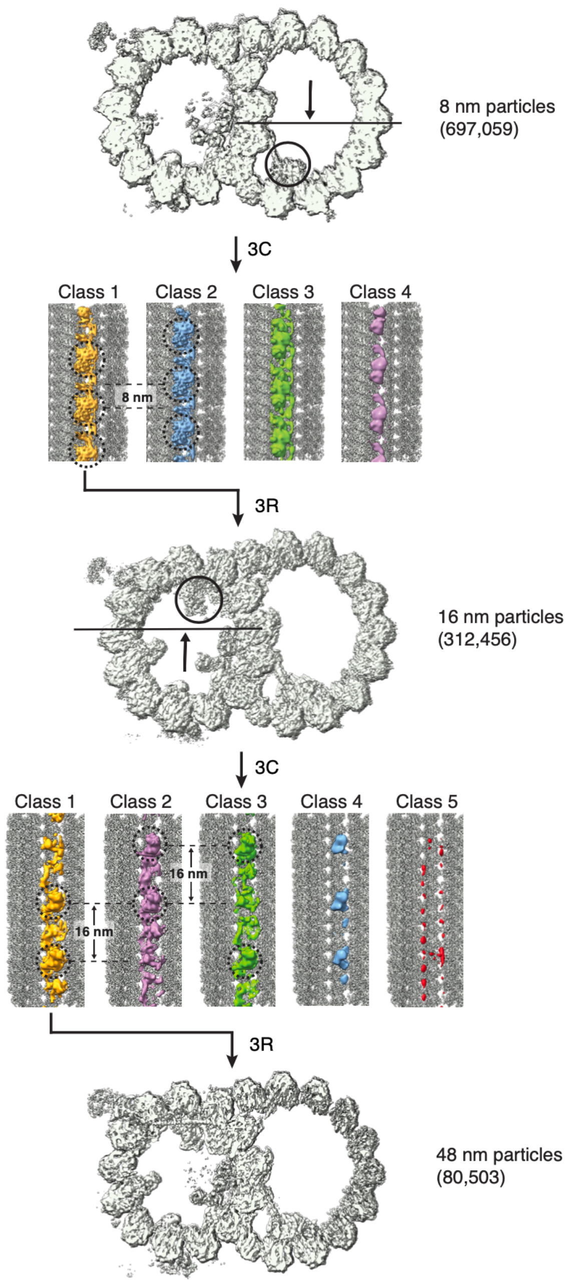

Processing Scheme 1. 8 to 48 nm. The 8-nm particles (*n* = 697,059) were classified into four classes using a cylindric mask (indicated by a circle) over the microtubule inner proteins at the inner junction. The most populous class, corresponding to the 16-nm repeat, was selected for further analysis. The 16-nm particles (*n* = 312,456) were classified into five classes using a cylindric mask (indicated by a circle) over the microtubule inner proteins at the seam of the A tubule. The most populous class, corresponding to the 48-nm repeat (n = 80,503) was selected for further analysis. Tubulin density was subtracted before classification.

Processing scheme 2: 48 to 96 nm

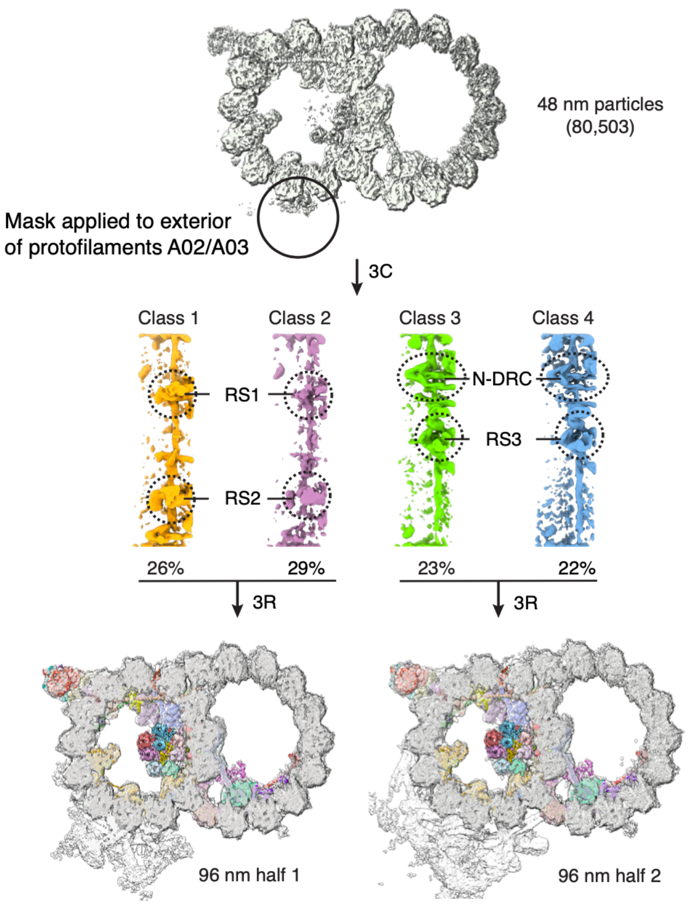

Processing Scheme 1. 48 to 96 nm. To determine maps corresponding to the two halves of the 96-nm repeat, the 48-nm particles (n = 80,503) were classified into four classes using a cylindric mask (indicted by a circle) over the external axonemal complexes bound to protofilaments A02 and A03. Classes were isolated corresponding to both halves of the 96-nm repeat; ones with radial spokes 1 and 2 (RS1 and RS2) and the others with RS3 and the nexin-dynein regulatory complex (N-DRC). Similar classes were combined and refined. Tubulin density was subtracted before classification.

Processing scheme ③: Determining a map of the ODA

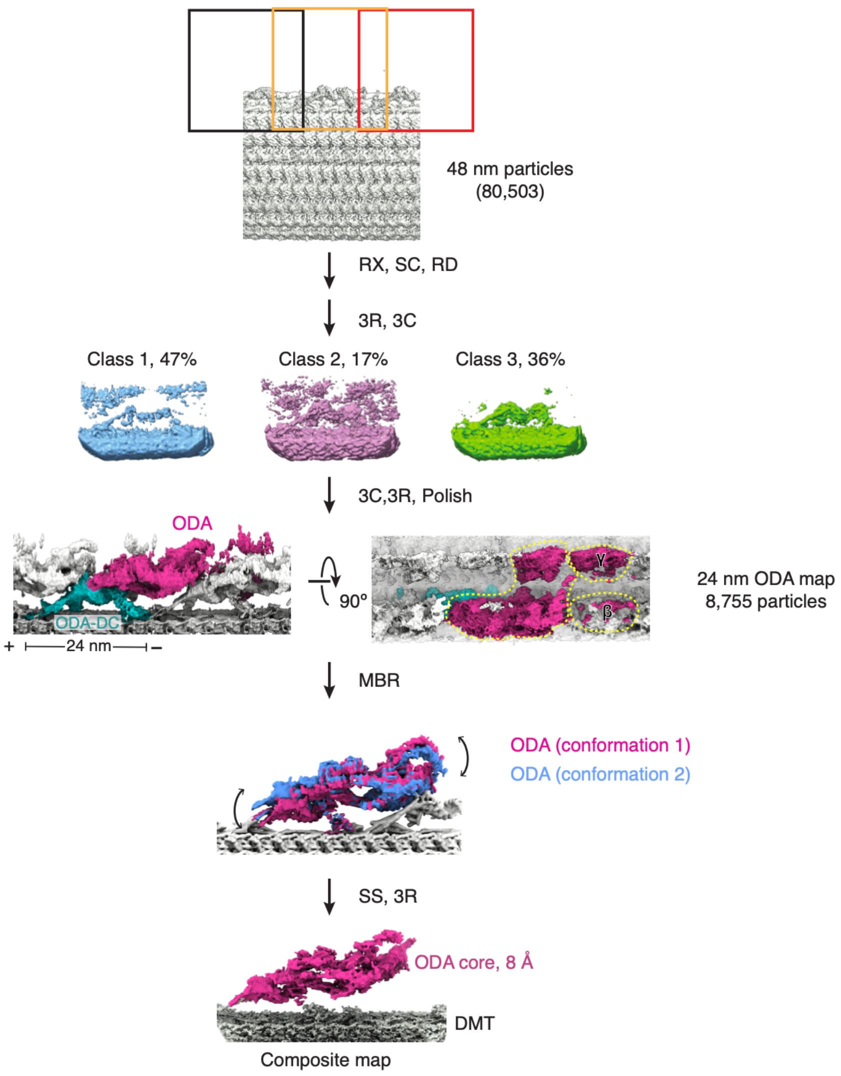

Processing scheme 3. Determining a map of the ODA. Processing scheme used to isolate ODA-bound particles. The 48-nm repeat map was divided into 3 subregions each focused on a full or partial copy of the ODA-DC. These regions were reboxed with a box size of 488 pixels and shifted to a common center. After excluding duplicates, the particles were aligned and reclassified to identify those with bound ODAs. The class with best density for the ODA was further classified, refined and polished. The motor domains of the β and γ subunits are poorly resolved compared to the ODA core due to flexibility and, for the γ subunit, averaging together paralogous heavy chains (DNAH11 and DNAH9) that localize to proximal to distal regions of the axoneme, respectively. Multi-body analysis of the remaining 8,755 particles revealed different conformations of the ODA relative to the doublet microtubule. The core of the ODA was refined, resulted in an 8-Å resolution reconstruction.

Processing scheme 4 : Lattice classification

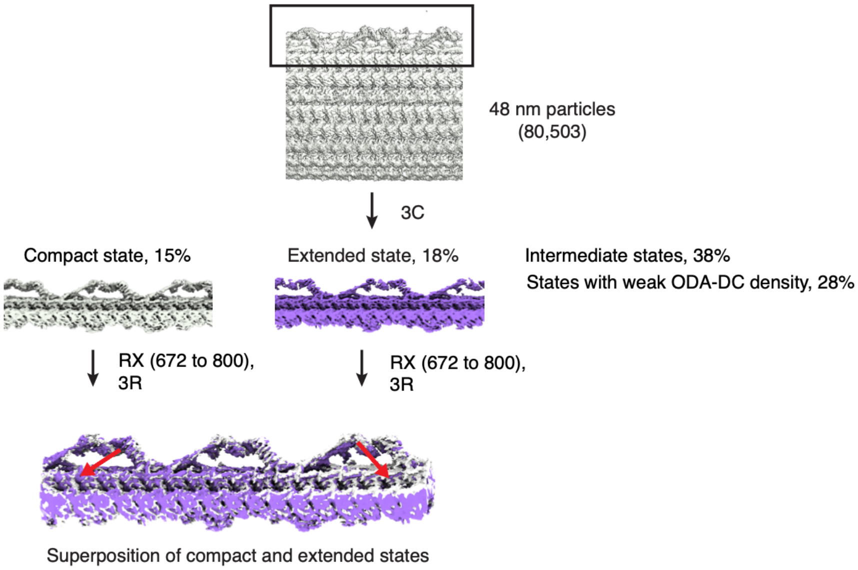

Processing scheme 4. Lattice Classification. Processing scheme used to isolate particles with different tubulin lattice dimensions. A cylindric mask was applied to classify protofilaments A07 and A08 into 8 classes. Particles with the shortest longitudinal spacing between tubulin heterodimers (the compact class) and the longest longitudinal spacing (the extended class) were re-extracted with a larger box size (800 pixels) and independently refined.

Processing scheme 5 : Tektin classification

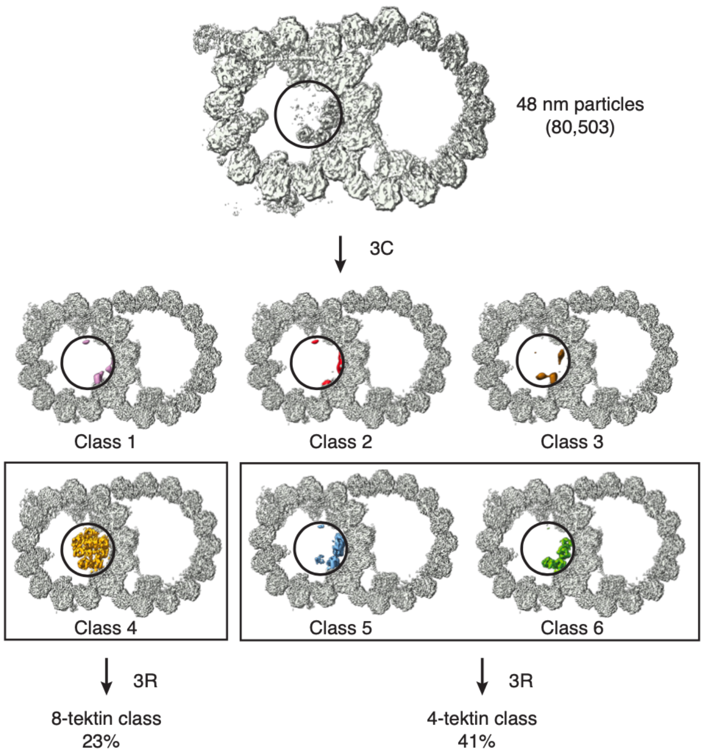

Processing scheme 5. Tektin classification. Density for the tektin bundle at the ribbon of the A tubule was less well-defined than other regions of the 48-nm map, indicating heterogeneity. We therefore performed focused classification using a cylindric mask (indicated by a circle) over the bundle. Classes were isolated corresponding to no tektin, 4 tektin filaments, and 8 tektin filaments. Classes containing 4- or 8-tektin bundles were selected and independently refined.

Processing scheme 6: Focused protofilament refinement/classification

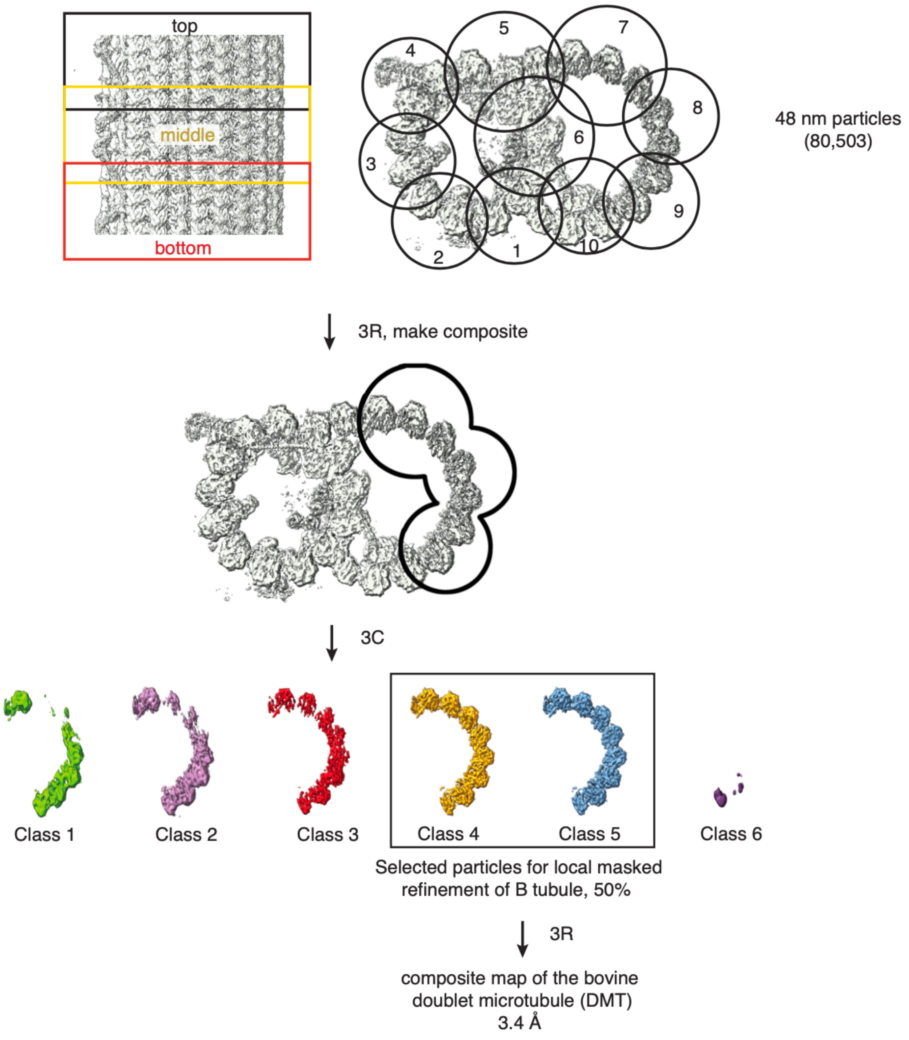

Processing scheme 6. Focused protofilament refinement/classification. To improve local map quality of the 48-nm repeat map, we performed a series of local refinements. Using a set of cylindric masks, the doublet microtubule was split into 10 overlapping subregions each containing 2 or 3 protofilaments. Each subregion was subdivided into 3 longitudinal sections (top, middle, and bottom) and refined separately. Additionally, we noticed that the density for protofilaments B02-B05 was poorly resolved. To improve the density of the B tubule, cylindric masks 7-9 were combined and used for focused three-dimensional classification. Classes displaying well-resolved density for protofilaments B02-B08 were merged and refined. This map is a constitute of the composite map of the bovine doublet microtubule.

Processing scheme 7: Determining a map of the ODA-DC

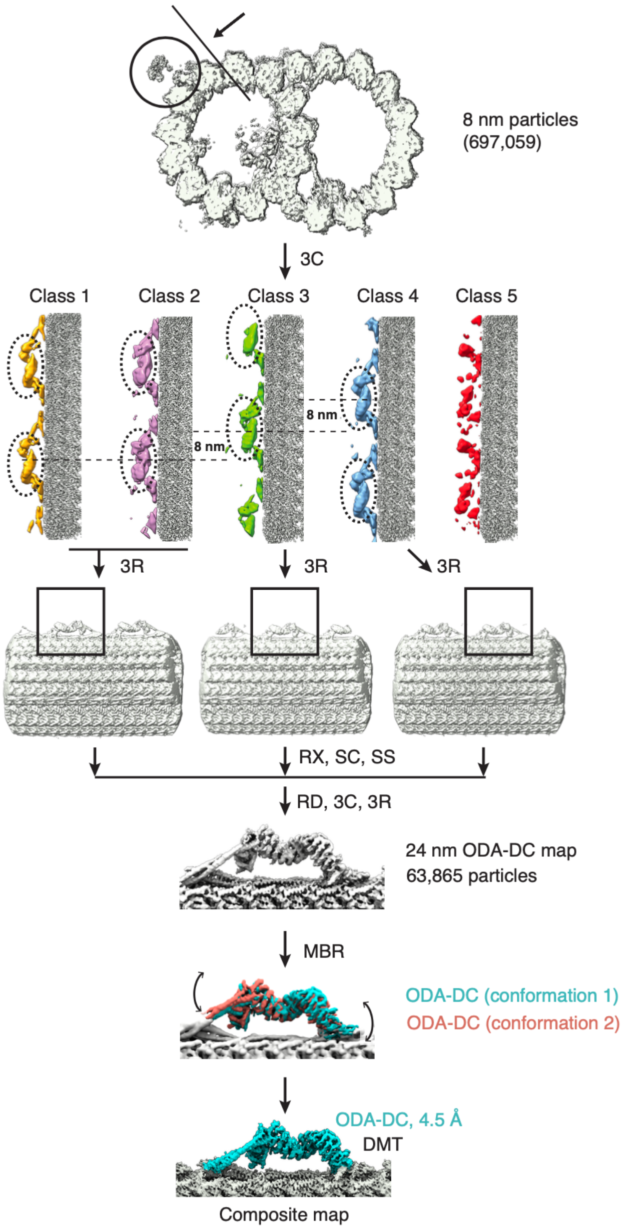

Processing scheme 7. Determining a map of the ODA-DC. Processing scheme used to generate a map of the ODA-DC. A cylindric mask was used to classify density corresponding to the ODA-DC apparent on protofilaments A07-A08 of the 8-nm repeat map. Classes corresponding to all three different possible registers of the ODA-DC were independently refined and combined with a box size of 288 pixels. Duplicates were excluded and the particles reclassified and refined. The particles were then subjected to multi-body refinement leading to a 3.6 Å resolution map of the ODA-DC-bound microtubule and a 4.5 Å resolution map of the ODA-DC.

Processing scheme 8: Determining maps of Pierce1 and Pierce2

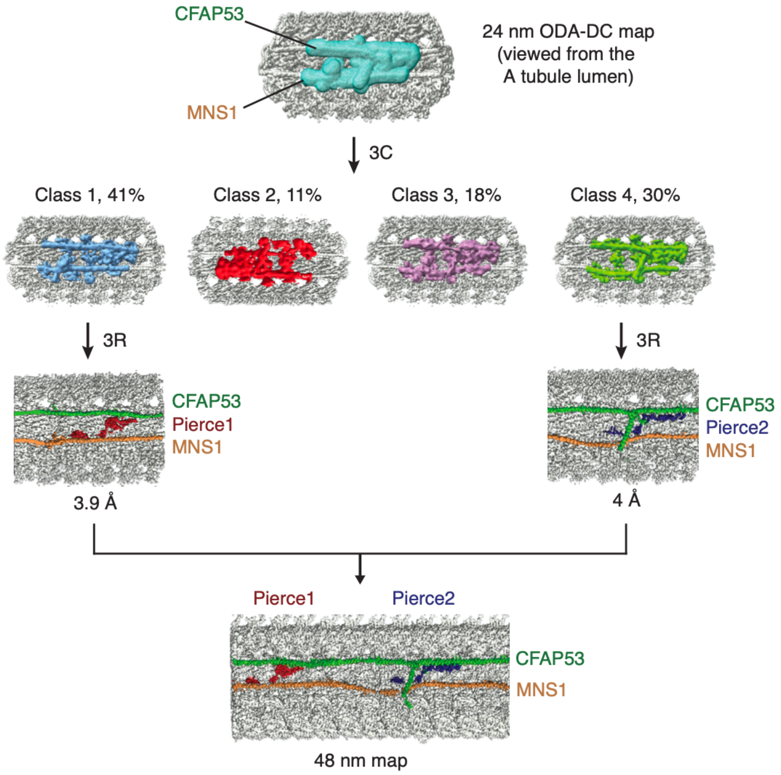

Processing scheme 8. Determining maps of Pierce1 and Pierce2. Processing scheme used to improve the map quality of Pierce1 and Pierce2. A shape mask was applied to MIP density on protofilaments A07-A09 of the 24-nm repeat map. Three-dimensional classification isolated two major classes: one centered on Pierce1 (left) and one centered on Pierce2 (right). These maps were independently refined and combined to generate the 48-nm repeat.

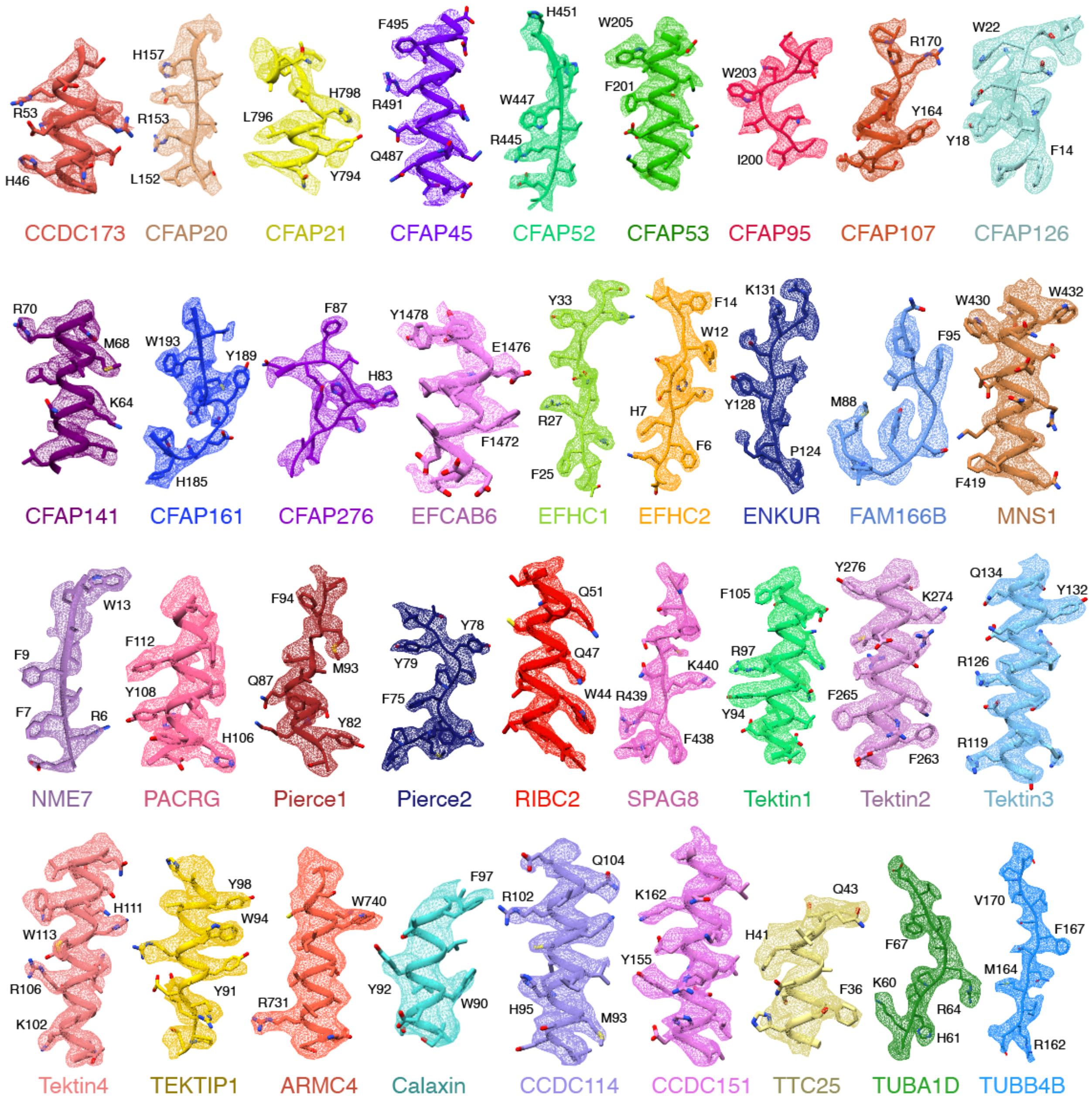

Examples of map quality for all identified proteins. Cryo-EM density for modeled regions of the doublet microtubule. Models are shown in stick representation within the density. Landmark residues are labeled. Densities are contoured at 4.5 – 7σ.

Proteins of the bovine DMT identified using cryo-EM.

Bovine axonemal protein	UniProt ID	*Chlamydomonas* ortholog	Molecular mass in Da / (number of residues)	Periodicity	Protofilament location	Built residues#	Percentage of residues built (%)
**ODA-DC**							
ARMC4 (ODAD2)	E1B8W3	-	115,859 (1044)	24 nm	A07-A08	448-1043	57
Calaxin (EFCAB1, ODAD5)	Q32L26	-	24,635 (212)	24 nm	A07-A08	4-34, 49-203	88
CCDC114 (ODAD1)	F1N2N9	DC2	77,563 (687)	24 nm	A07-A08	28-277	36
CCDC151 (ODAD3)	A7MBH5	DC1	72,044 (621)	24 nm	A07-A08	68-316	40
TTC25 (ODAD4)	A5PK42	-	78,368 (683)	24 nm	A07-A08	12-129, 185-258	28
**Luminal proteins (MIPs)**							
CFAP20	Q6B857	FAP20	22,748 (193)	8 nm	Inner junction	1-184	95
CFAP21 (EFHB)	F1MMV1	FAP21	97,819 (877)	48 nm	A08-A10	291-492, 585-607, 608-674, 710-755, 760-813, 814-865	51
CFAP45*	Q32LN4	FAP45	65,666 (549)	48 nm	B07-B10	75-92, 101-150, 159-543	83
CFAP52 (WDR16)	E1BKF9	FAP52	68,637 (623)	16 nm	B09-B10	13-622	98
CFAP53 (MBD1, CCDC11)	F1N7G5	FAP53	62,015 (514)	48 nm	A06-A11	35-122, 123-507	92
CFAP95 (C9orf135)	Q32L77	FAP95	26,623 (232)	48 nm	A09-A11	89-103, 112-153, 154-217	52
CFAP107 (C1orf158)	Q2TA11	FAP107	23,234 (196)	48 nm	A09-A12	6-196	97
CFAP126	Q3SZT6	FAP126	21,185 (196)	16 nm	A12-A13	2-119	60
CFAP141 (C1orf189)	Q32L75	FAP141	12,339 (101)	48 nm	A09-A11	11-101	90
CFAP161 (C15orf26)	F6RJC2	FAP161	36,470 (321)	2 copies every 48 nm	A09-A11	4-97, 111-271	79
CFAP210 (CCDC173)	E1BJL9	FAP210	65,300 (547)	48 nm	B06-B10	38-90, 91-170, 177-300, 307-328, 336-493	80
CFAP276 (C1orf194)	E1B9I5	FAP276	19,358 (170)	16 nm	B09-10, A13-A01	66-101,128-170	46
EFCAB6†	A0A4W2DZI5	-	172,742 (1,497)	48 nm	A13-B10	1319-1496	12
EFHC1	E1BKH1	RIB72	74,032 (640)	16 nm	A12-A05	7-54, 84-369, 414-531	71

EFHC2	A0A3Q1N1R0	RIB72	85,454 (733)	16 nm	A12-A05	2-188, 200-293, 302-365, 374-402, 418-596, 602-684	87
ENKUR (CFAP106)	E1B836	FAP106	30,157 (259)	16 nm	A12-13, B09-B10	9-95, 106-253	91
FAM166B	Q2TBR5	-	30,514 (274)	8 nm	A01-A05	14-64, 84-116, 172-208, 234-264	55
MNS1	F1MH18	FAP127	60,456 (494)	48 nm	A07-A11	130-483	72
NME7	Q5E9Y9	FAP67	42,599 (377)	2 copies every 48 nm	A09	4-375	99
PACRG	A5PK71	PACRG	29,284 (257)	8 nm	Inner junction	38-256	85
Pierce1 (C9orf116)	Q32P67	FAP182	15,654 (136)	48 nm	A07-A08	22-136	85
Pierce2 (C15orf65)	A0A3Q1LFK7	FAP182	13,771 (120)	48 nm	A07-A08	25-120	80
RIBC2	Q32LJ7	RIB43a	44,695 (377)	48 nm	A11-A13	5-377	99
SPAG8	E1BNS6	FAP143	51,542 (484)	48 nm	A09-A13	286-300, 309-370, 383-452	30
Tektin 1	Q32KZ9	-	48,719 (418)	16 nm	A tubule lumen	6-403	95
Tektin 2	Q2T9Q6	-	49,886 (430)	16 nm	A tubule lumen	2-428	99
Tektin 3‡	A6H782	-	56,681 (490)	16 nm	A tubule lumen	43-486	91
Tektin 4	Q2TA38	-	51,945 (447)	16 nm	A tubule lumen	22-447	95
TEKTIP1 (C19orf71)	Q2M2T2	-	24,500 (208)	16 nm	Associated with tektins	15-158	69

# Underscored residues have sidechains truncated due to the map quality.

* Unable to differentiate three CFAP45 isoforms (UniProt entries Q32LN4, F6PXG2, and A0A3Q1MJ67) by density or mass spectrometry.

† Unable to differentiate between UniProt entry A0A4W2DZI5 from *Bos indicus* x *Bos taurus* (Hybrid cattle) and NCBI entry XP_003586209.2, which has three amino acids different from A0A4W2DZI5. An alternative EF-hand-containing protein cannot be discounted at this position.

‡ Unable to differentiate two Tektin 3 isoforms (UniProt entries A6H782 and F1MX07) by density or mass spectrometry.

In all cases of ambiguity, the UniProt entry listed in the table above was used for modeling.
