# Supplementary material for: De novo identification of mammalian ciliary motility proteins using cryo-EM
Source: Cell. Author manuscript; Available in PMC 2021 Nov 17. (PMC8595878; doi:10.1016/j.cell.2021.10.007)
Supplement: 14 [file NIHMS1749474-supplement-14.pdf]

CellPress
OPEN ACCESS

Supplemental figures

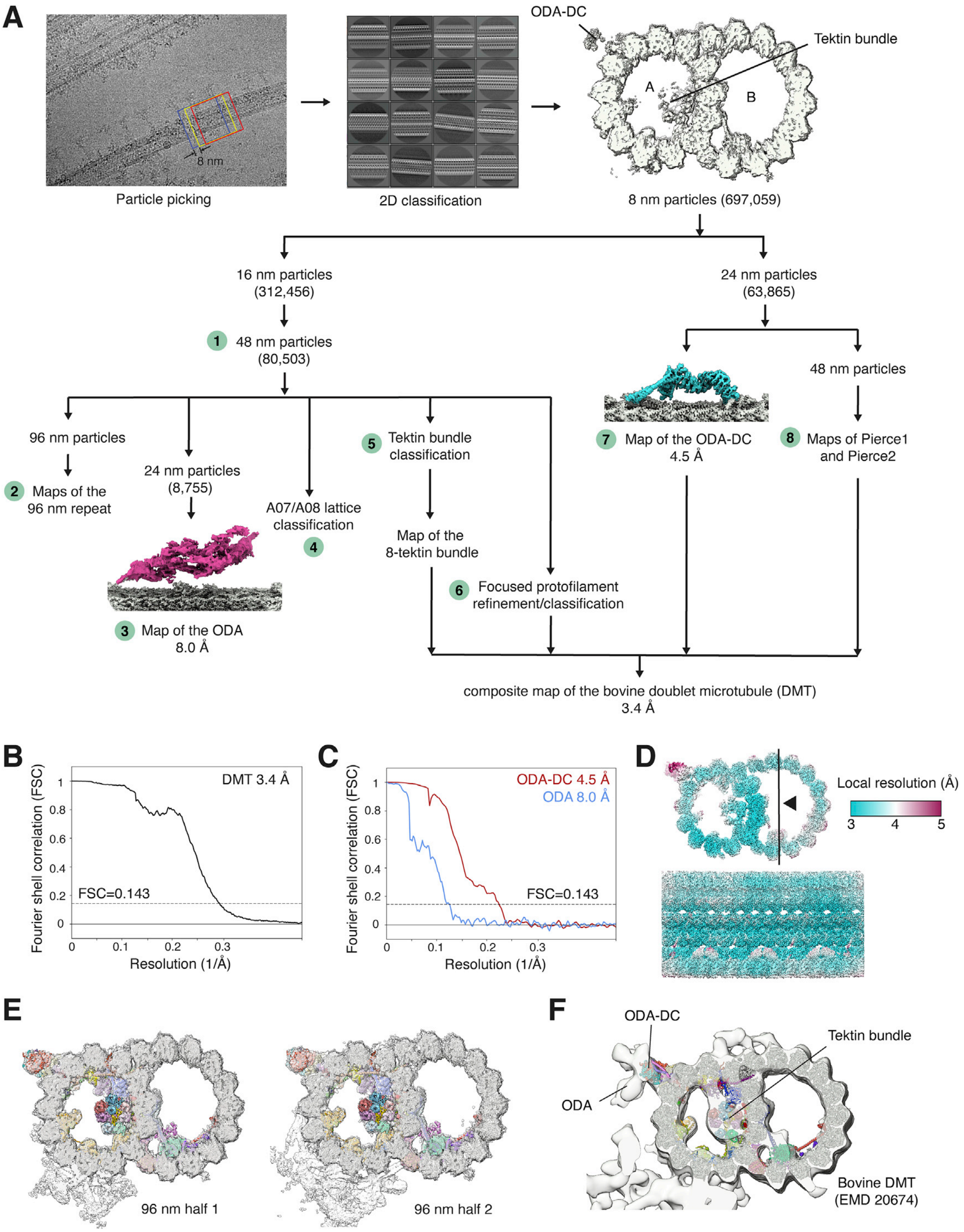

Figure S1. Cryo-EM data processing, related to Figure 1

A. Simplified flow diagram showing the processing strategies used to determine structures of the bovine DMT with and without bound ODAs. Processing starts with extracting particles every 8 nm along the long axis of the DMT. The 8-nm particles are sorted by two-dimensional classification, with selected class averages

(legend continued on next page)

shown, before three-dimensional refinement to generate a map of the 8-nm repeat of the bovine DMTs. The particles (n = 697,059) contributing to this map are used for all subsequent processing steps. Each processing strategy is indicated with a number and is described in more detail in Methods S1.

B. Fourier Shell Correlation (FSC) curve for the final cryo-EM map of the bovine DMT (DMT). The intersection of the horizontal dashed line (at 0.143) with the FSC curve indicates the nominal resolution of the map.

C. FSC curves for the ODA-DC and ODA.

D. Composite map of the DMT colored by local resolution.

E. Atomic model of the bovine DMT docked into both halves of the 96-nm repeat.

F. Atomic model of the bovine DMT docked into the subtomogram average of the bovine DMT.

CellPress
OPEN ACCESS

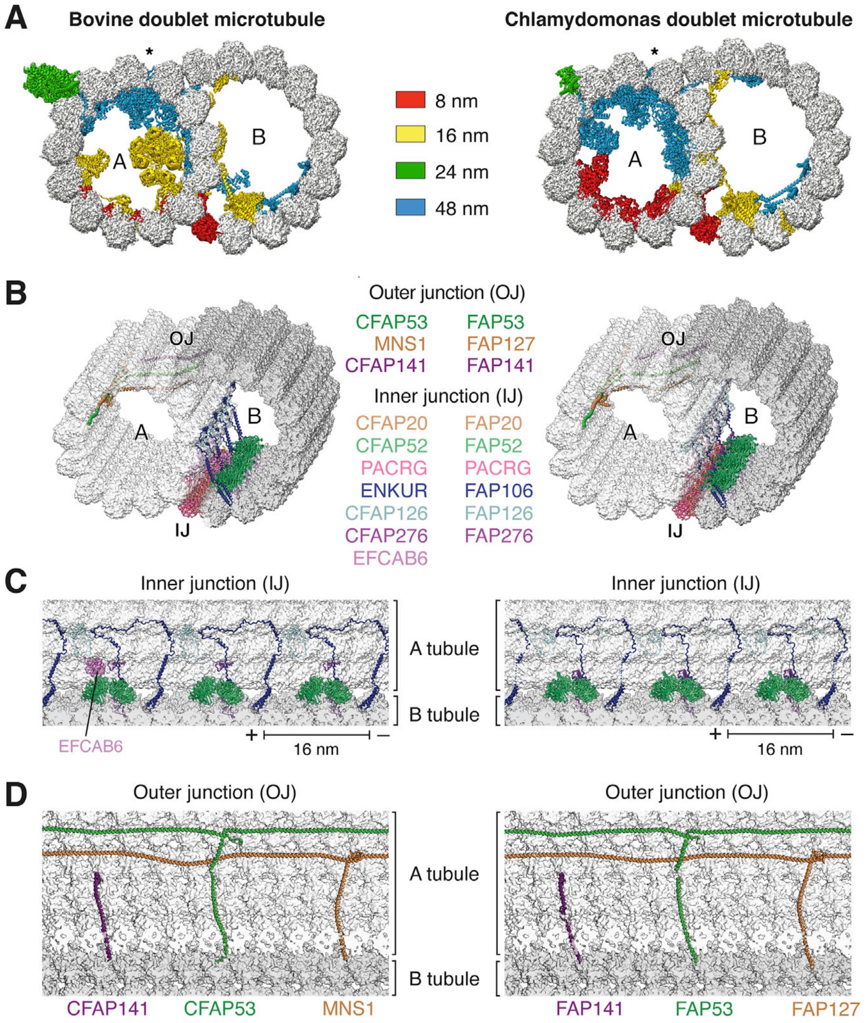

Figure S2. Conservation of MIPs across mammalian and algal DMTs, related to Figure 1

In all panels, bovine DMTs (this study) are shown on the left and *Chlamydomonas* DMTs (PDB 6U42) (Ma et al., 2019) are shown on the right.

A. Slice through bovine and *Chlamydomonas* DMTs showing non-tubulin proteins colored by periodicity. Proteins with 24-nm periodicity are restricted to the exterior whereas MIPs can have 8, 16 and 48-nm repeat lengths. In *Chlamydomonas*, MIPs with 16-nm periodicity are predominantly distributed in the B tubule. The seam of the A tubule marked with an asterisk

B. Comparison of MIPs at the inner (IJ) and outer junction (OJ). Bovine DMTs have an additional MIP, EFCAB6, at the inner junction. The names of shared MIPs are given for both *Bovine* and *C. reinhardtii*.

C. Longitudinal view showing the arrangement of MIPs at the inner junction. The position of EFCAB6 in the bovine DMT is indicated.

D. Longitudinal section showing the three helical MIPs that span the walls of the A tubule to directly contact the B tubule.

Proteins in panels B, C and D have uniform colors. The minus (-) and plus (+) ends of the longitudinal sections are indicated next to the scale bar.

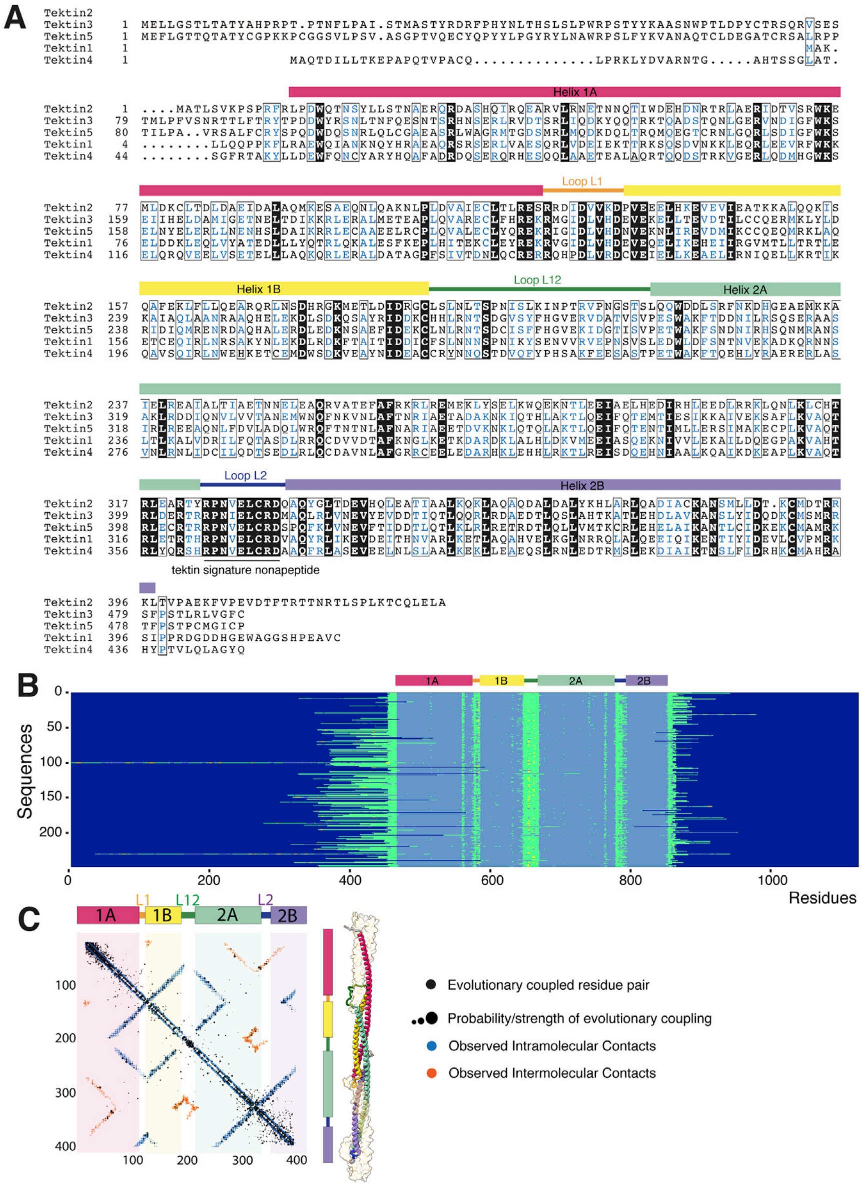

Figure S3. Sequence and structure conservation of tektins, related to Figure 3

A. Alignment of tektin paralogs from *Bovine*. Secondary structure is shown above the alignment.

B. Conservation of helix length across tektin homologs. Secondary structure prediction of 249 tektin sequences. Residues predicted to be in α helices are in light blue, loops in green, and β sheets in yellow. Dark blue denotes end of the sequence's residues. The sequences are aligned on the highly conserved L2 loop.

C. Conservation of the tektin fold revealed by evolutionary coupling (EC). Coevolving residues pairs with a probability greater than 40% are plotted together with the observed intra- and intermolecular residue contacts for Tektin 1.

CellPress
OPEN ACCESS

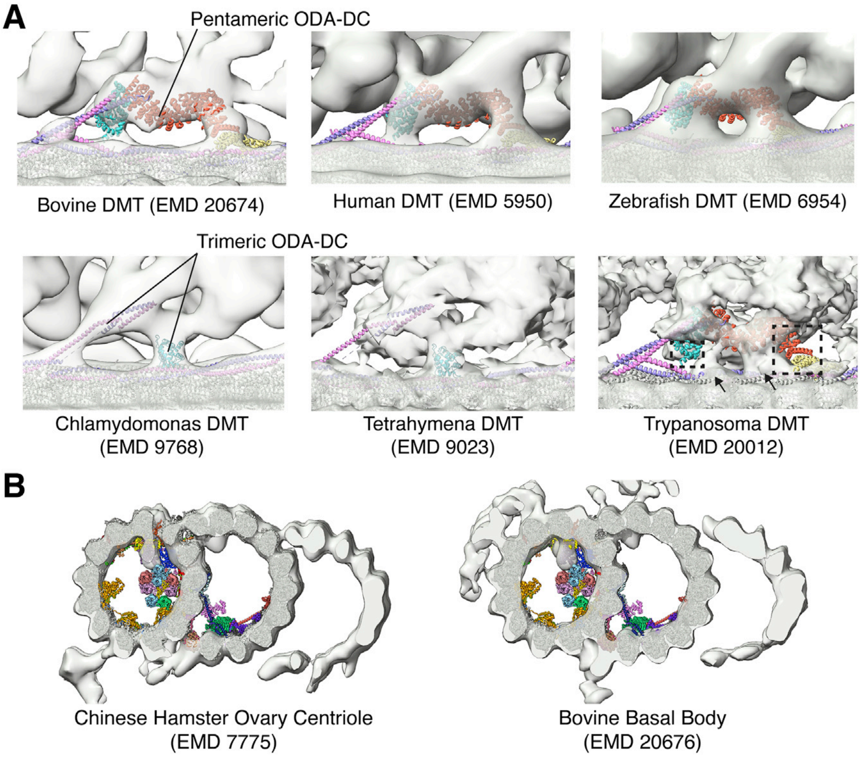

Figure S4. Comparison of the bovine tracheal DMT model with subtomogram averages, related to Figures 3 and 4

A. Atomic models of the bovine pentameric ODA-DC (this study) and the *Chlamydomonas* trimeric ODA-DC (PDB 7KZO) docked into subtomogram averages of DMTs from various organisms. The fit of the atomic model was evaluated by docking both the pentameric and trimeric models into the subtomogram averages. The pentameric ODA-DC model is consistent with subtomogram averages from bovine, human, and zebrafish axonemes. The trimeric ODA-DC model is consistent with subtomogram averages from *Chlamydomonas* and *Tetrahymena* axonemes. Neither model sufficiently explains the density observed in the *Trypanosoma* subtomogram average. For *Trypanosoma,* the pentameric model does not explain all density observed (arrows) in the subtomogram average, while portions of the model fall outside the subtomogram average density (dashed boxes).

B. Model of the bovine DMT docked into the subtomogram average of the centriole obtained from the Chinese Hamster Ovary (CHO) cell line (left) and the basal body obtained from bovine trachea (right). Density consistent with tektin is not observed in either centriole or basal body.

For all panels the accession code of the subtomogram average in the Electron Microscopy Data Bank (EMDB) is given in parentheses.

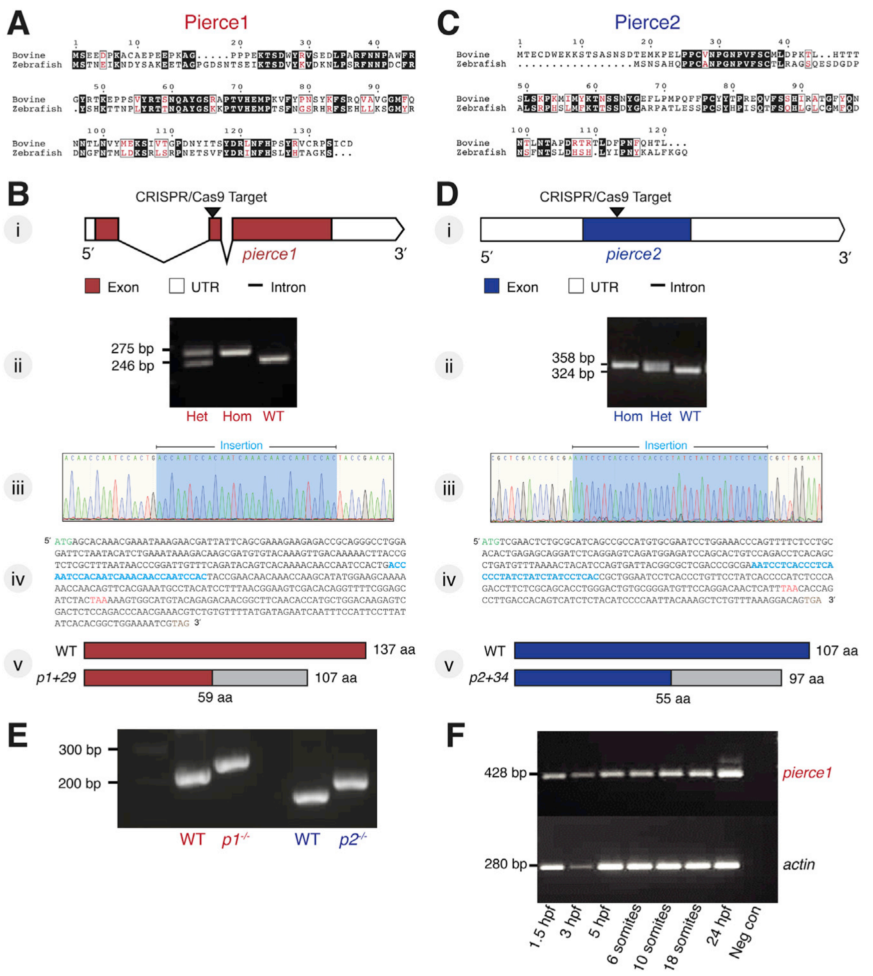

Figure S5. Generation of zebrafish mutants of *pierce1* and *pierce2*, related to Figure 6

A. Sequence alignment of the bovine and zebrafish homologs of Pierce1. Conserved residues are boxed and colored red. Identical residues are highlighted in black.

B. Generation of zebrafish mutants of *pierce1*. From top to bottom: (i) Schematic illustration of the zebrafish *pierce1* locus. The CRISPR/Cas9 target site is within exon 2 of the *pierce1* gene. (ii) *pierce1* transcripts amplified by PCR. A larger product was observed in heterozygous and homozygous embryos relative to WT, representing the mutant allele. (iii) Sequencing chromatogram of PCR products confirmed a 29-nucleotide insertion in the mutant allele. (iv) Position of the insertion in the *pierce1* gene. (v) The 29-nucleotide insertion leads to disruption of the protein sequence after residue 59 and premature termination relative to WT.

C. Sequence alignment of zebrafish and bovine Pierce2. Conserved residues are boxed and colored red. Identical residues are highlighted in black.

D. Generation of zebrafish mutants of *pierce2*. From top to bottom: (i) Schematic illustration of the zebrafish *pierce2* locus. The CRISPR/Cas9 target site is within the single exon of the *pierce2* gene. (ii) *pierce2* transcripts amplified by PCR from heterozygous, homozygous and WT embryos. A larger product was observed in heterozygous and homozygous embryos, representing the mutant allele. (iii) Sequencing chromatogram of PCR products confirmed a 34-nucleotide insertion in the mutant allele. (iv) Position of the insertion in the *pierce2* gene. (v) The 34-nucleotide insertion leads to disruption of the protein sequence after residue 55 and premature termination relative to WT.

E. Reverse transcription PCR confirms that both the $p1^{-/-}$ and $p2^{-/-}$ mutations persist in the respective mRNAs of $p1^{-/-}$;$p2^{-/-}$ double knockout zebrafish.

F. cDNA was made from zebrafish embryos at 1.5 hpf, 3 hpf, 5 hpf, 6 somites, 10 somites, 18 somites and 24 hpf. PCR for *pierce1* and *actin* (loading control) was carried out on these cDNAs. *pierce1* transcripts were identified from the earliest developmental time-point indicating maternal contribution of *pierce1* mRNA.

CellPress
OPEN ACCESS

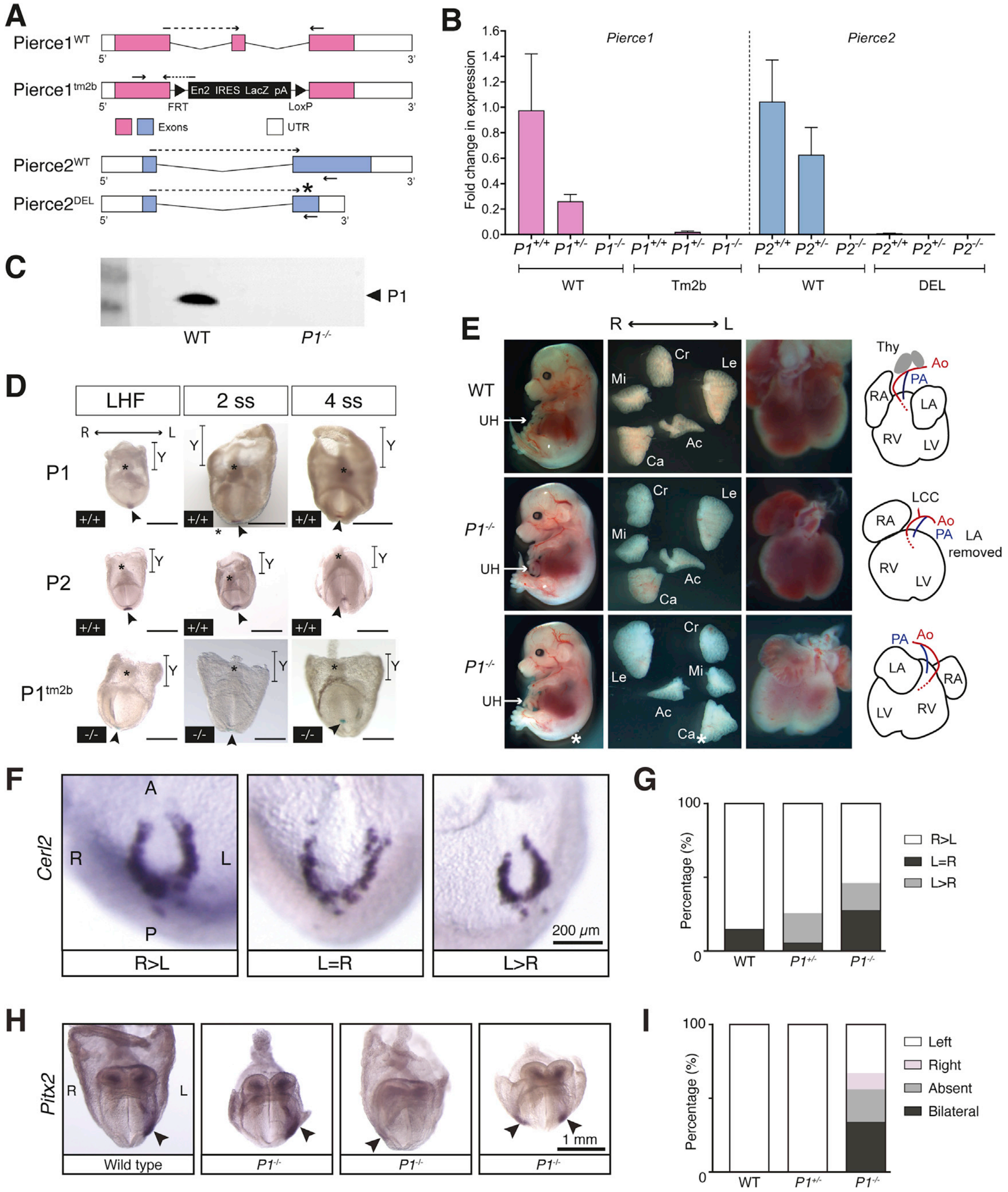

Figure S6. Pierce1-deficient embryos display *situs* abnormalities, related to Figure 7

A. Mouse allele generation. The *Pierce1*^{tm2b} allele is a gene trap generated using the Cre-LoxP system and contains an En2 splice acceptor, IRES and LacZ reporter in the place of exon 2. This is expected to result in the expression of truncated Pierce1 (P1) protein, with LacZ expressed from the same transcript (p.Y80SfsTer12). The *Pierce2*^{DEL} allele is a CRISPR-generated deletion of 321 nt that is predicted to result in a premature termination codon (NM_001198789.1:c.296_616del). Arrows are positions of RT-qPCR primers, dotted lines are primers which span exon-exon junctions, asterisk is region of CRISPR deletion.

B. Validation of *P1* and *P2* allele mRNA expression in adult mouse testes. *P1*^{tm2b} and *P2*^{DEL} allele mRNA expression cannot be detected. Pink bars = *P1* testes, blue = *P2* testes. Primers used to detect the respective alleles are below genotype. Error bars represent standard deviation (SD).

C. Validation of P1 protein loss from mouse testes. Total lysate from WT and *P1*$^{-/-}$ mice testes were probed with anti-P1 antibody. A band representing P1 was detected at 20 kDa in WT but not *P1*$^{-/-}$ testes. The blot is representative of two replicates.

D. P1 and P2 are expressed in the node of E8.0-E8.5 mouse embryos. Top two panels show WISH data for *P1* and *P2* in WT embryos, whereas the lower panel depicts LacZ staining in *P1*$^{-/-}$ embryos. LHF = late head fold, ss = somite stage, scale bars = 1 mm. Arrows indicate nodes, asterisks show position of each allantois, and Y = yolk sac.

(legend continued on next page)

E. Photographs of E14.5 mouse embryos, their lung lobation and position of the heart outflow tracts. Unmarked display *situs solitus*, asterisks indicate *situs inversus totalis*. Diagrams to the right show heart morphology for easier comparison. UH = physiological umbilical hernia, Cr = cranial lung lobe, Mi = middle lung lobe, Ca = caudal lung lobe, Le = left lung lobe, Acc = accessory lung lobe, Thy = thymus, RA = right atrium, LA = left atrium, RV = right ventricle, LV = left ventricle, LCC = left common carotid artery, Ao = aorta (red), PA = pulmonary artery (blue).

F. *Cerl2 in situ* hybridization patterns observed in $P1^{+/+}$, $P1^{+/-}$ and $P1^{-/-}$ embryos at E8.0-E8.5. The anterior (A), posterior (P) and left (L) and right (R) axes are annotated. Scale bar = 200 μm.

G. Quantification of *Cerl2* expression. $P1^{-/-}$ embryos exhibited aberrant *Cerl2* gene expression.

H. *Pitx2* expression in WT and in $P1^{-/-}$ mouse embryos at E8.0-E8.5. Stained areas are highlighted with arrows, and the left and right sides of the embryo are annotated. Scale bar = 1 mm.

I. Quantification of *Pitx2* expression. $P1^{-/-}$ embryos exhibited aberrant *Pitx2* gene expression.
